# Imputation Free Deep Survival Prediction with Conditional Variational Autoencoders

**DOI:** 10.1007/s41666-026-00234-y

**Published:** 2026-04-10

**Authors:** Natalia Hong, Aditya Acharya, Krishna Gokhale, Jenny Cooper, Charles Gadd, Francesca Crowe, Krishnarajah Nirantharakumar, Christopher Yau

**Affiliations:** 1 Nuffield Department of Women’s & Reproductive Health, University of Oxford, Oxford, United Kingdom; 2 Health Data Research UK, London, United Kingdom; 3 The Alan Turing Institute, London, United Kingdom; 4 Department of Applied Health Sciences, University of Birmingham, Birmingham, United Kingdom; 5 Department of Population Health Sciences, Kings College London, London, United Kingdom

**Keywords:** Missing data, Electronic health records, Survival prediction, Deep learning, Variational autoencoder

## Abstract

Electronic Health Records (EHRs) provide rich opportunities for developing risk prediction tools to support clinical decision-making, yet they are inherently incomplete because data are recorded selectively during routine care. Such missingness may be informative, reflecting clinical judgment and patient status, and missing data patterns can shift between model development and real-world deployment. These challenges limit the reliability and transportability of predictive models in healthcare settings. We propose an imputation-free framework that jointly trains Conditional Variational Autoencoders with deep survival models to enable risk prediction directly from incomplete EHR data. We demonstrate the approach using the deep survival model DeSurv and evaluate its performance through simulation studies and two retrospective cohorts from the Clinical Practice Research Datalink primary care database. The proposed framework consistently outperforms conventional missing data methods, achieving superior performance on ground-truth metrics in simulations and improved calibration-based survival metrics in real-world cohorts. It also demonstrates increased robustness to unseen missingness patterns and distributional shifts. By providing a unified strategy for handling missing data across development, validation, and deployment, this work advances methodological robustness in healthcare informatics and supports more reliable clinical risk prediction in practice.

**Supplementary Information:**

The online version contains supplementary material available at 10.1007/s41666-026-00234-y.

## Introduction

The adoption of Electronic Health Records (EHRs), primarily designed to improve patient management by digitising health data, also enables secondary uses in research. However, using EHRs retrospectively for research poses challenges, including the inherent incompleteness of these records. These gaps in data do not necessarily reflect deficiencies in data quality but rather the nature of clinical workflows and selective recording. Clinicians order tests based on individual symptoms and clinical needs [1], with patients experiencing severe conditions often having more comprehensive records through frequent visits and tests [2, 3], whereas underserved groups may have sparser EHRs due to limited healthcare access [4].Consequently, the absence of measurements can carry implicit information about a patient’s condition, making missingness itself potentially informative of outcomes [5]. Therefore, consideration must be given to how patient interactions with the healthcare system influence the information recorded in EHR, and data gaps must not be misinterpreted as random [6].

Incomplete EHR is commonly approached as a missing data problem, with methods like imputation applied without careful scrutiny [7]. Imputation is a process where missing values are estimated to create complete datasets for model fitting, and requires validating a secondary imputation model under untestable missingness mechanism assumptions. Effectively applying these methods requires understanding the distinction between predictive modelling, which emphasises accurate forecasting of outcomes, and inferential modelling, which focuses on uncovering unbiased relationships within the data [8]. This distinction is important as predictive models can exploit missingness patterns that carry predictive information [9] and, unlike inferential models, can encounter missing data at test time [5, 10]. This contrast is reflected in greater emphasis in the literature on inferential modelling, where imputation aims to preserve data relationships and usually assumes that missingness can be explained by all observed data, including the outcome [11]. This assumption rarely holds in EHR, and in predictive modelling, imputation must rely exclusively on observed predictors since the outcome is unknown. Transportability poses another challenge, requiring imputation models developed during training to be deployable and maintainable. For example, QRISK3 [12], a cardiovascular risk prediction model, uses Multiple Imputation by Chained Equations (MICE) with outcome during *development* but transitions to a simpler mean imputation based on age and sex at *deployment*, highlighting the challenges of using complex imputation strategies [5].

Pattern submodels are an alternative approach to imputation by creating submodels for each missingness pattern, while extensions, such as the sharing pattern submodel, enables information sharing across submodels [13, 14]. While these methods handle outcome-related missingness, i.e. missingness that correlates with outcome, without assuming a missingness mechanism, straightforward implementations scale poorly with the exponential growth of missing patterns and are computationally impractical for large datasets. Moreover, the sharing pattern submodel is limited to linear models, restricting its use in modern deep learning frameworks.

Our work builds upon the pattern submodel, introducing an imputation-free framework for survival prediction. This utilises Conditional Variational Autoencoders (cVAEs) combined with deep survival models to directly predict risk from incomplete data, without assuming a missingness mechanism. The framework does not require cached imputation models or developmental data at deployment, ensuring consistency between training and deployment and preserving the validity of model evaluations. The framework learns the distribution of missingness patterns within the VAE latent space, capturing similarities in a regularised manner, and integrates the resulting latent embeddings into the deep survival model, enabling non-linear modelling while avoiding combinatorial inefficiencies. This approach is validated through simulation studies and retrospective cohorts from the CPRD database, demonstrating improved performance in generalising to unseen missingness patterns and to missingness shifts.

**Fig. 1:**
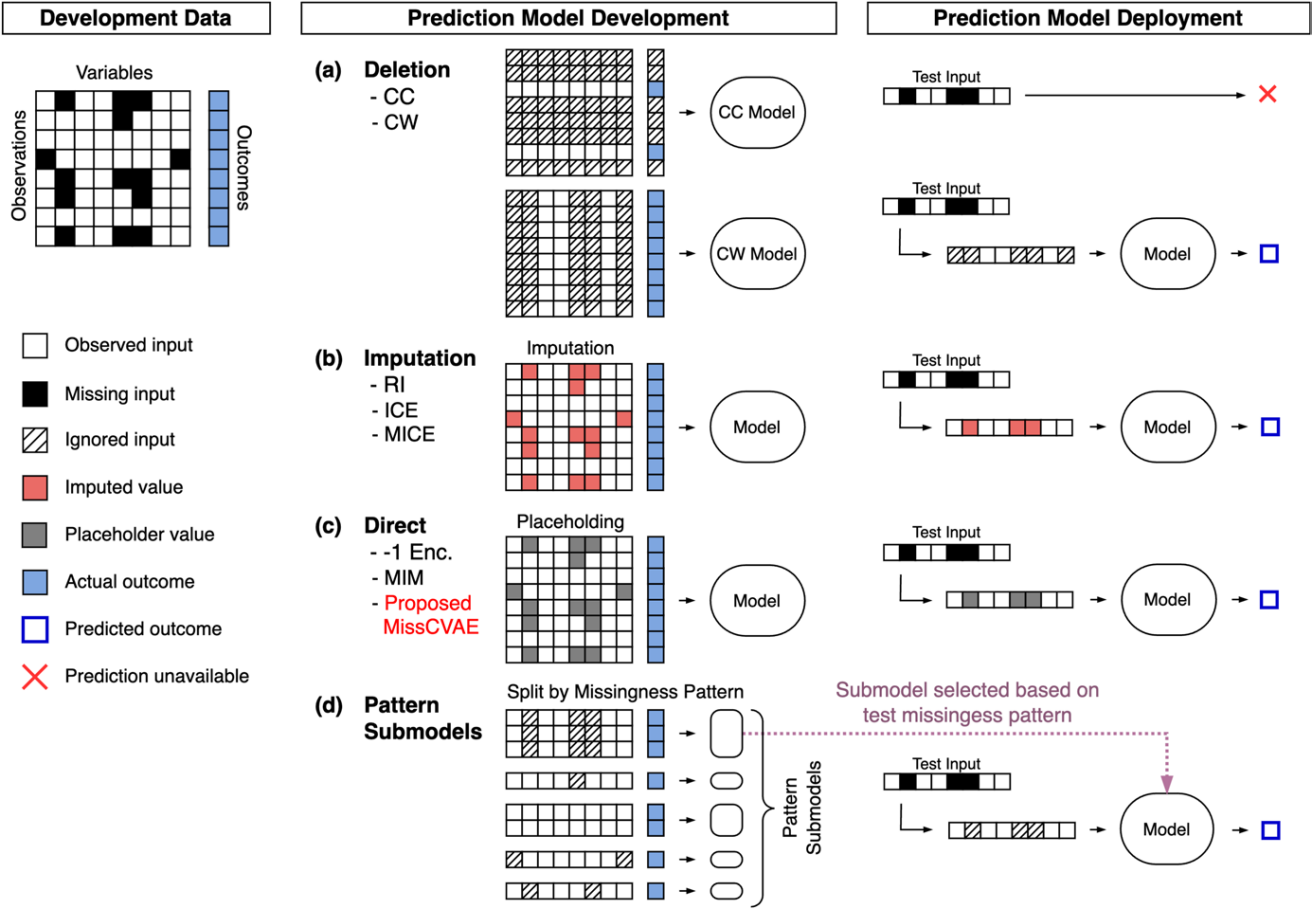
Illustration of missing data strategies in prediction models

**Table 1:** Missing data strategies in prediction models

Method	Exploit Outcome Related	Deployment Feasibility
	Missingness for Prediction	
CC	$\times$	Limited to complete test data
CW	$\times$	Limited to observed variables
RI	$\times$	Supports deployment
ICE	$\times$	Supports deployment
MICE	$\times$	Requires re-fitting to development dataset
-1 Enc.	$\checkmark$	Supports deployment
MIM	$\checkmark$	Supports deployment
PS	$\checkmark$	Limited to seen missingness patterns
**MissCVAE**	$\checkmark$	Supports deployment

## Related Work

In Fig. 1 and Table 1, we summarise various strategies for handling missing time-to-event data in prediction modelling, excluding those specific to binary or continuous outcomes beyond our scope. These strategies include approaches like deletion methods, imputation-based techniques, direct methods, and pattern submodels.

Deletion methods create complete datasets by removing rows (listwise deletion, or complete-case, CC) or columns (column-wise deletion, CW) containing missing values. While CC can be efficient, it often results in substantial data loss and relies on the strong assumption that data are Missing Completely at Random (MCAR), meaning missingness occurs purely by chance. CW deletion is useful for excluding irrelevant variables, but it can weaken model performance if important predictors are omitted.

Imputation methods aim to generate sensible estimates for missing values, thereby creating complete datasets. Univariate imputation methods replace missing values with simple summaries such as the mean, median, or mode, but these approaches ignore inter-variable relationships. In contrast, multivariate imputation methods leverage the correlations among variables to generate more accurate estimates. For instance, Regression Imputation (RI) builds predictive models for each variable using the observed data, while Imputation by Chained Equations (ICE) iteratively refines estimates by cycling through variables, treating each as a dependent variable and using updated imputations as predictors until convergence [15]. Multiple Imputation by Chained Equations (MICE) [16, 17], widely regarded as the standard approach for inference, generates multiple imputed datasets to capture the uncertainty inherent in the imputation process. However, MICE can be computationally prohibitive for high-dimensional data with significant missingness and is not suitable for test-time imputation. This is because it requires refitting the imputation model by combining new and original data, which is often constrained by data access limitations [8, 18]. Additionally, the MICE R package does not store intermediate imputation model coefficients, as they are theoretically invalidated by the iterative and sequential nature of the imputation process [19]. Rubin’s rules [16], commonly used to combine parameter estimates from models fitted on each imputed dataset in statistical analyses, also cannot be applied to deep learning models, as neural network weights cannot be directly averaged.

Direct methods address missing entries by substituting them with placeholders, rather than attempting to reconstruct the missing data. For example, if the data is normalised to [0, 1], an out-of-range constant such as –1 can be used (–1 Enc.). The Missing Indicator Method (MIM) augments the dataset with binary indicators for missing entries, filling the missing entries themselves with a placeholder (like zero) or imputed values. This allows models to directly learn from missingness patterns, but can lead to overfitting in high-dimensional datasets by overly relying on these patterns [20]. While MIM can be unsuitable for Cox regression due to interpretability challenges, it generally performs well with flexible, non-linear models by accommodating diverse missingness patterns, though the increased dimensionality can introduce instability.

Pattern submodels instead fit separate models to data subsets defined by missingness patterns, bypassing the need for imputation or explicit assumptions about the missingness mechanism [13, 21]. This approach can handle outcome-related missingness, but independent models do not share information resulting in suboptimal data usage, particularly when many patterns are present or the patterns are sparse. Sharing Pattern Submodels address this limitation by enabling parameter sharing across submodels, though this is restricted to linear models [14]. Both approaches, however, cannot handle unseen missingness patterns, as they lack the ability to generate predictions for cases that do not match any of the pre-defined trained submodels.

**Fig. 2:**
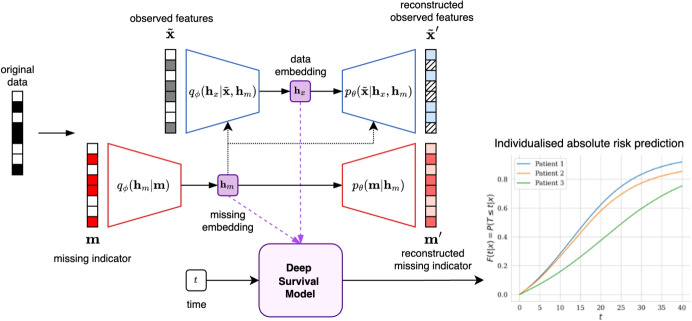
**MissCVAE** Architecture. Observed and missing data vectors are separately encoded into latent variables which feed into the deep learning-based survival prediction model

## Proposed Framework: MissCVAE

Survival methods model time-to-event, often assuming non-informative censoring, where censoring is independent of event time and subject characteristics. Let ${\textbf {x}}$ be a *p*-dimensional random vector of risk factors and let ${\textbf {m}}\in \{0,1\}^p$ indicate missingness, where $m_j = 1$ if $x_j$ is observed and 0 if missing. Let $\textbf{x}^{\text {obs}}$ denote the subset of entries of $\textbf{x}$ for which $m_j = 1$. We define the observed feature vector as $\tilde{\textbf{x}}$ such that

$$\tilde{x}_j = {\left\{ \begin{array}{ll} x_j, & m_j = 1\\ \text {NA}, & m_j = 0 \end{array}\right. },$$

where NA marks unobserved entries. Thus, $\tilde{\textbf{x}}$ preserves the observed values $\textbf{x}^{\text {obs}}$ in their original positions while explicitly encoding missing values as NA. The dual survival outcome $(T, \delta )$ comprises *T*, the event or censoring time, and $\delta$, and indicator where $\delta = 1$ denotes event occurrence and $\delta = 0$ denotes censoring, indicating the subject did not experience the event within the observation window. The event time is described by the cumulative distribution function $F(t) = \mathbb {P}(T \le t)$, the corresponding probability density function *f*(*t*), and the survival function $S(t) = \mathbb {P}(T> t) = 1 - F(t)$, which gives the probability of surviving past time *t*.

Modern survival models directly estimate survival probabilities without assuming proportional hazards, with deep learning approaches framing it as a mapping:

$$S(t|{\textbf {x}}) = \varphi (f_{\gamma }(t, {\textbf {x}})),$$

where $f_{\gamma }$ is a function given by a deep learning model parameterised by $\gamma$, and $\varphi$ is a link function mapping the output to $[0, 1]$, ensuring interpretability as a probability. These models typically maximise the right-censored likelihood under the assumption of independent censoring,

$$L = \prod _{i=1}^n f(t_i|{\textbf {x}}_i)^{1\!\!1(\delta _i=1)} S(t_i|{\textbf {x}}_i)^{1\!\!1(\delta _i=0)},$$

where an observed event contributes the density at event time, and a censored observation contributes the survival at censoring time.

Our proposed **MissCVAE** approach extends this framework by incorporating mechanisms to handle missingness, as illustrated in Fig. 2. The framework employs a deep latent variable model to generate embeddings for both $\tilde{\textbf{x}}$ and $\textbf{m}$, which are then used as inputs in place of the original features for survival prediction. Conceptually, our estimand of interest is the survival function conditional on the observed covariates and their missingness pattern, $S(t \mid \textbf{x}^{\text {obs}}, \textbf{m})$. Operationally, we encode $(\textbf{x}^{\text {obs}}, \textbf{m})$ via $(\tilde{\textbf{x}}, \textbf{m})$ and parameterise the survival function as

$$\begin{aligned} S(t \mid \tilde{\textbf{x}}, \textbf{m}) = \varphi \!\left( f_{\gamma }\big (t, \{\textbf{h}_x, \textbf{h}_m\}\big )\right) , \end{aligned}$$

where $\textbf{h}_x$ and $\textbf{h}_m$ are learned latent embeddings of $\tilde{\textbf{x}}$ and $\textbf{m}$, respectively. We use $p_\theta (\tilde{{\textbf {x}}}, {\textbf {m}}) = p_\theta (\tilde{{\textbf {x}}}, {\textbf {m}}| {\textbf {h}}_x, {\textbf {h}}_m) p_\theta ({\textbf {h}}_x, {\textbf {h}}_m)$, in which the observed data $\tilde{{\textbf {x}}}$ and missingness pattern ${\textbf {m}}$ are generated from latent variables ${\textbf {h}}_x$ (data embedding) and ${\textbf {h}}_m$ (missing embedding). Specifically, we consider the following setup:

$$\begin{aligned} {\textbf {h}}_m&\sim \text {Normal}( 0, {\textbf {I}}), \\ {\textbf {h}}_x&\sim \text {Normal}( 0, {\textbf {I}}) , \\ {\textbf {m}}&\sim \text {Bernoulli}(\mathcal {D}^{m}_{\theta }({\textbf {h}}_m)), \\ \tilde{{\textbf {x}}}&\sim \text {Normal}(\mathcal {D}^{x}_{\theta }({\textbf {h}}_x, {\textbf {h}}_m)), \end{aligned}$$

where $\mathcal {D}^{m}_{\theta }$ and $\mathcal {D}^{x}_{\theta }$ are decoder networks parameterised by $\theta$. Here, $\mathcal {D}^{m}_{\theta }$ outputs the mean and variance parameters of the Normal distribution. We apply amortised variational inference to approximate the posterior distribution $p_\theta ({\textbf {h}}_x, {\textbf {h}}_m | \tilde{{\textbf {x}}}, {\textbf {m}})$ by a family of variational distributions $\{q_\phi ({\textbf {h}}_x, {\textbf {h}}_m | \tilde{{\textbf {x}}}, {\textbf {m}}) \}_\phi$ using

$$\begin{aligned} {\textbf {h}}_m | {\textbf {m}}&\sim \text {Normal}( \mathcal {E}^m_{\phi }({\textbf {m}})), \\ {\textbf {h}}_x | \tilde{{\textbf {x}}}, {\textbf {h}}_m&\sim \text {Normal}(\mathcal {E}^x_{\phi }(\tilde{{\textbf {x}}}, {\textbf {h}}_m) ), \end{aligned}$$

where $\mathcal {E}^m_{\phi }$ and $\mathcal {E}^x_{\phi }$ are encoder networks parameterised by $\phi$, outputting the mean and variance paramters of the Normal distribution.

This architecture is a two-tiered VAE-cVAE [22]. First, discrete binary missingness indicators are mapped to a continuous embedding space using a secondary VAE encoder that captures the distribution of missingness patterns. These missing embeddings serve as auxiliary inputs to the primary cVAE, which models the conditional distribution of the data given the missingness pattern. Both the missing and data embeddings are then used as inputs to a survival prediction model. The overall training objective combines the VAE loss with the survival likelihood, enabling the embeddings to accurately represent the input data while being predictive of survival outcomes. This joint training optimises the evidence lower bound on the log-likelihood (ELBO), given by:

$$\begin{aligned}&\log p_{\theta }({\textbf {x}}^{\text {obs}}, {\textbf {m}}, {\textbf {t}}) \\&= \log \int p_{\theta }({\textbf {x}}^{\text {obs}}, {\textbf {m}}, {\textbf {t}}, {\textbf {h}}_x, {\textbf {h}}_m) \;\text {d}{\textbf {h}}_x \text {d}{\textbf {h}}_m\\&\ge \underbrace{\mathbb {E}_{{\textbf {h}}_x, {\textbf {h}}_m \sim q_{\phi }} \left[ \log p_{\theta }({\textbf {t}}|{\textbf {h}}_x, {\textbf {h}}_m)\right] }_{\text {Survival Likelihood}} \\&\quad \underbrace{+\mathbb {E}_{{\textbf {h}}_x, {\textbf {h}}_m \sim q_{\phi }} \left[ \log p_{\theta }({\textbf {x}}^{\text {obs}}| {\textbf {h}}_x, {\textbf {h}}_m) + \log p_{\theta }({\textbf {m}}| {\textbf {h}}_m)\right] }_{\text {Reconstruction}}\\&\quad \underbrace{ \begin{aligned}&- \mathbb {E}_{{\textbf {h}}_m \sim q_{\phi }} \left[ \text {KL}\left( q_\phi ({\textbf {h}}_x|{\textbf {h}}_m, {\textbf {x}}^{\text {obs}}) || p_{\theta }({\textbf {h}}_x)\right) \right] \\&-\text {KL}\left( q_\phi ({\textbf {h}}_m|{\textbf {m}}) || p_{\theta }({\textbf {h}}_m)\right) \end{aligned} }_{\text {KL Regularisation}} \end{aligned}$$

and the full derivation is provided in Supplementary Section II.

As imputation is not the focus, reconstruction loss is computed only on observed values ${\textbf {x}}^{\text {obs}}$. To improve prediction and prevent Kullback-Leibler (KL) divergence vanishing, objective function components can be weighted to balance reconstruction, regularization, and survival prediction.

While VAEs have been previously suggested to handle missing data, they are typically applied in the context of imputation within unsupervised settings. A few exceptions integrate imputation with downstream prediction, bridging missing data handling and predictive modelling [23]. In contrast, our approach de-emphasises imputation, focusing on prediction directly.

### Relationship with Pattern Submodels

Pattern submodels create distinct submodels $f_{C(\textbf{m})}$ for each categorical pattern, where $C(\textbf{m}) = 1 + \sum _{j=1}^p 2^{p-j}m_j$ encodes the unique combination of missingness indicators. The sharing pattern submodels improves this by enabling information sharing across submodels via shared parameters in linear models. Both rely on explicit handling of each pattern which limits scalability. Instead of separate submodels, a cVAE conditions the latent representation on categorical patterns $C(\textbf{m})$, enabling a single model to predict survival probabilities. This approach is scalable, leverages the flexibility of non-linear networks, and shares information across patterns. However, its dependence on dense categories may limit generalisation to rare or unseen missingness patterns.

Our framework extends the cVAE by integrating a probabilistic representation of missingness patterns via an auxiliary VAE, which learns the structure of missingness and generates missing embeddings to enhance the primary cVAE’s conditional distribution. This improves generalisation to rare patterns and regularises the influence of missingness indicators, unlike -1 Enc or MIM. Our framework therefore offers a scalable and robust approach to handling complex missingness scenarios.

**Fig. 3:**
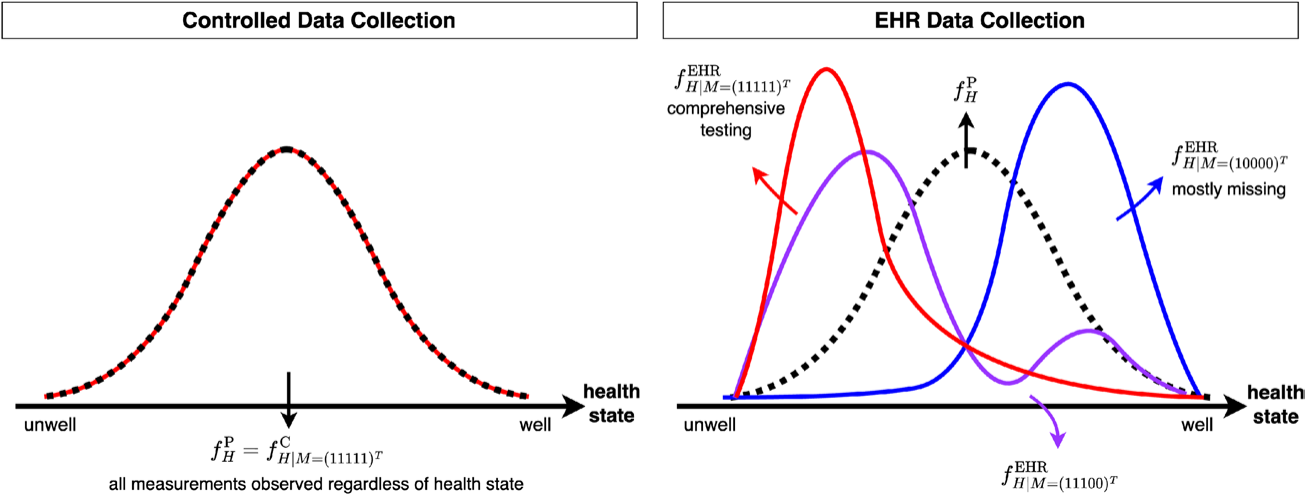
Example distributions or probability density function (PDF) of the latent health state under different missingness patterns. Here, $f_H^{\text {P}}$ represents the population PDF of the health state. The conditional sample PDFs, $f_{H|M}^{\text {C}}$ and $f_{H|M}^{\text {EHR}}$, represent latent health distributions conditioned on missingness patterns in controlled and EHR data, respectively

## Experiments

### Simulation

**Fig. 4:**
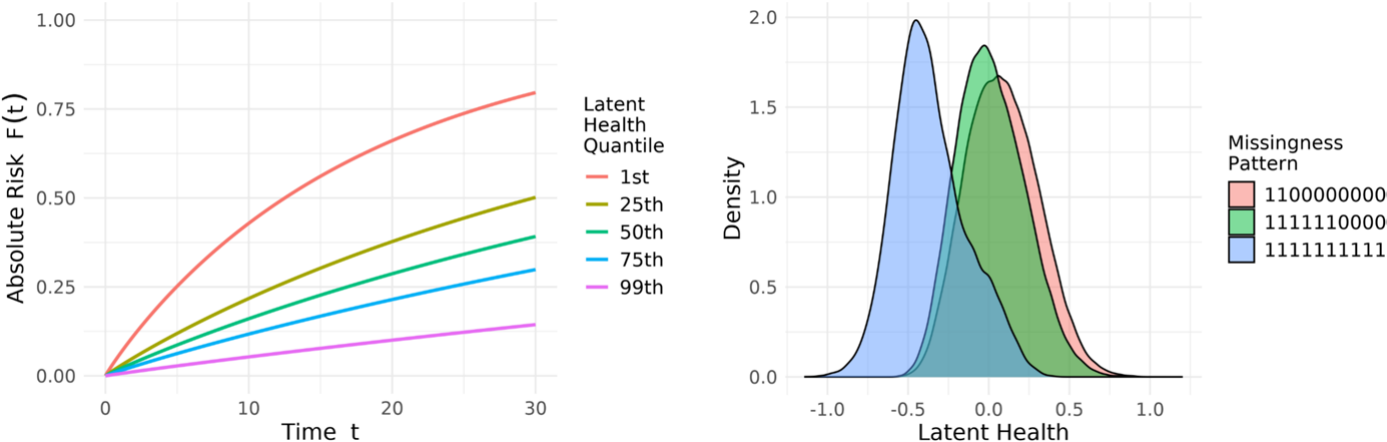
Example simulation output where a latent health variable is simulated, with higher values indicating healthier patients, influencing both outcome and data completeness. (Left) Five absolute risk curves corresponding to five quantiles of latent health. (Right) Density distributions of the latent health variable stratified by three distinct missingness patterns represented as a binary string, where ‘1’ denotes an observed variable and ‘0’ denotes a missing variable

We conduct simulations to address the challenges of using real-world EHRs to evaluate new methodologies. Unlike controlled data collection, which is designed to systematically capture information across a defined range of health conditions, EHRs may reflect patterns of missingness that are influenced by underlying health status or healthcare utilisation. This could result in an observed distribution that, when conditioned on missingness patterns, deviates from the true population distribution (Fig. 3). As the underlying mechanisms driving missingness in EHRs cannot be precisely identified, simulations provide the ability to define and control these mechanisms, enabling systematic evaluation of model performance. In addition, in survival analysis, real-world data provides observed event indicators but lacks the true underlying survival probabilities for each individual. Consequently, survival metrics often evaluate only specific aspects of performance, relying on event indicators and reweighting methods. Simulated data with predefined survival distributions enables precise error quantification by directly comparing predicted survival curves to true probabilities. We simulate data using latent variables that drive the risk factor, outcome and missingness generation, capturing real-world dynamics where unobservable factors like health status or lifestyle behaviours affect not only the outcome but also data completeness, thereby reflecting the Missing Not At Random (MNAR) mechanisms [16] in EHRs. The simulation follows a multi-step framework:

1. **Simulate Latent Variables**: Simulate *d* latent variables $H=(H_1,...,H_d)$ from a multivariate Normal distribution:
  $$H \sim \mathcal {N}(\mu _H, \Sigma _H).$$
  For example, $H_1$ may represent latent health state, with larger values indicating better health.
2. **Simulate Risk Factors**: A set risk factors, $X=(X_1, \dots , X_p )$, each simulated independently conditioned on latent variables from Step 1, inducing correlations through their shared dependence:
  $$X_j = f_j(H) \quad j = 1, \dots , p.$$
  For example, systolic blood pressure can be simulated from $\mathcal {N}(130-20H_1, 25)$, increasing as the latent health worsens (Supplementary Table 1).
3. **Simulate Survival Outcomes**: Using the variables from Steps 1 and 2, event times $T_E$ are simulated using a proportional hazards model via inverse sampling
  $$\lambda (T_E \mid H, X) = \lambda _0(T_E) \exp (\beta _H^\top H + \beta _X^\top X),$$
  and censoring times $T_C$ are independently sampled from an exponential distribution. The observed time *T* and event indicator $\delta$ are defined as:
  $$T = \min (T_E, T_C), \quad \delta = 1(T_E \le T_C).$$
  Parameters should be consistent with prior steps, such as ensuring that poorer health is associated with worse outcomes (e.g., $\beta _{H_1} < 0$) (Supplementary Table 2).
4. **Induce Missingness**: To simulate realistic missingness patterns, we introduce a latent missingness variable *Z* with the same dimensionality as *X*:
  $$Z \sim \mathcal {N}(\mu _{\text {z}}, \Sigma _{\text {z}}).$$
  We define $\mu _{\text {z}} = f_{\mu }(H)$, where latent variables from Step 1 influence missingness (Supplementary Table 3). As outcomes are also derived from these latent variables, this naturally induces outcome-related missingness (Fig. 4). The covariance matrix $\Sigma _{\text {z}}$ encodes how the absence of one risk factor correlates with the absence of others and is represented as a sparse matrix to reflect joint missingness, where frequently co-observed measurements form clusters [24, 25]. For example:
  $$\Sigma _{\text {z}} = \begin{pmatrix} 1 & \quad x_1 & \quad & \quad & \quad & \quad & \quad & \quad & \quad & \quad \\ x_1 & \quad 1 & \quad & \quad & \quad & \quad & \quad & \quad & \quad & \quad \\ & \quad & \quad 1 & \quad y_1 & \quad y_2 & \quad y_3 & \quad & \quad & \quad & \quad \\ & \quad & \quad y_1 & \quad 1 & \quad y_4 & \quad y_5 & \quad & \quad & \quad & \quad \\ & \quad & \quad y_2 & \quad y_4 & \quad 1 & \quad y_6 & \quad & \quad & \quad & \quad \\ & \quad & \quad y_3 & \quad y_5 & \quad y_6 & \quad 1 & \quad & \quad & \quad & \quad \\ & \quad & \quad & \quad & \quad & \quad & \quad 1 & \quad z_1 & \quad z_2 & \quad z_3 \\ & \quad & \quad & \quad & \quad & \quad & \quad z_1 & \quad 1 & \quad z_4 & \quad z_5 \\ & \quad & \quad & \quad & \quad & \quad & \quad z_2 & \quad z_4 & \quad 1 & \quad z_6 \\ & \quad & \quad & \quad & \quad & \quad & \quad z_3 & \quad z_5 & \quad z_6 & \quad 1 \end{pmatrix}.$$
  Here, blanks are zeroes and off-diagonal entries represent correlations within clusters, such as groups of anthropometric measurements or routine tests.
  To induce missingness, a threshold is applied to *Z*
  $$M_j = 1(Z_j> 0), \quad j = 1, \dots , p,$$
  where $M_j = 1$ indicates that the *j*-th feature is observed, and $M_j = 0$ indicates it is missing.

We design three simulations (baseline, unseen missingness, missingness shift) of 50,000 samples each, using two latent variables, one representing the patient’s underlying health state and another for their lifestyle behaviour. Each simulation includes five fully observed risk factors and 15 partially observed ones, resulting in $2^{15} = 32,768$ possible missingness patterns. Full details are given in Supplementary Section III.

**Fig. 5:**
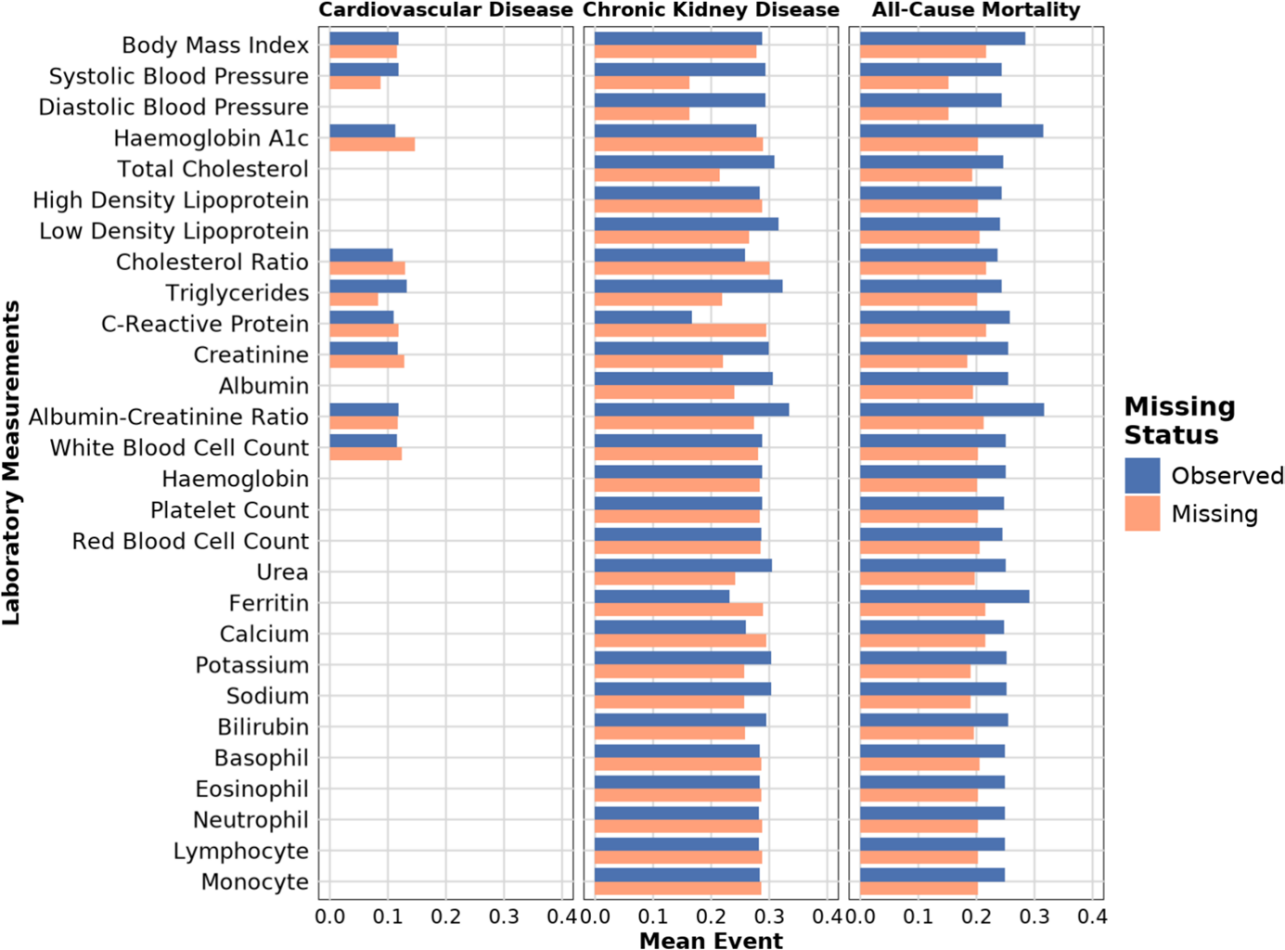
Mean event incidence for patients with missing versus observed values, shown separately for each laboratory measurement

### Real-World Primary Care Data

We also investigate performance using EHR from the CPRD Aurum database [26]. This comprises anonymised primary care data collected from general practices across the UK. Eligible participants for research are extracted using the Data Extraction for Epidemiological Research tool (DExtER) [27, 28], including participants that are actively registered after 1 January 2005 for at least 12 months (index date), with follow-up until 30 May 2022. From this dataset, we generate three cross-sectional datasets for our experiments. All datasets include complete risk factors such as sex, ethnicity, index of multiple deprivation (IMD), smoking, treatment indicators and prior conditions, and laboratory measurements. For patients with multiple assessments, values closest to the prediction reference date within an appropriate window are used.

The first dataset considers cardiovascular disease (CVD) prediction in individuals diagnosed with Type 2 diabetes (T2D) between ages 60 to 80 without prior CVD diagnosis, starting from the T2D diagnosis date [29, 30]. Only individuals whose T2D diagnosis occurred after their index date are included, resulting in a maximum follow-up of 17.5 years. The dataset includes 36,457 London-registered patients, a 9.1% incidence rate, and nine laboratory measurements recorded within [-365, +30 days] of T2D diagnosis. Figure 5 shows measurement observation proportions and CVD incidence for missing and observed measurements. Of $2^9=512$ possible missingness patterns, 306 are observed.

The second dataset studies Chronic Kidney Disease (CKD) stages 3–5 in individuals registered in London, aged 65–75 at their index dates, with at least two of the following conditions: hypertension, osteoarthritis, T2D, CVD, depression, anxiety, or cancer, resulting in a maximum follow-up period of 17.5 years. Twenty-eight laboratory measurements are extracted within a [-540, +30 days] window around their index date. This broader window is chosen as there is no direct diagnosis date to anchor measurements, necessitating a longer period to ensure more data availability. The dataset contains 77,175 observations with an incidence rate of 22.6%. Of $2^{28} = 268,435,456$ possible missingness patterns, 9,505 are observed.

The third dataset examines a broader non–disease-specific outcome, namely all-cause mortality (ACM) in individuals aged 65–70 on 1 January 2005, which serves as the prediction time zero. All individuals are registered in London, and follow-up continues until 31 December 2019, providing a maximum horizon of 15 years. As with the CKD dataset, twenty-eight laboratory measurements are collected within a [-540, +30]-day window around 1 January 2005. It contains 64,586 individuals with an ACM incidence rate of 21.8%. Of the $2^{28}$ possible missingness patterns, 4,440 are observed.

The full set of features and data summary is available in Supplementary Tables 4 and 5.

### Baseline and Metrics

The experiments are benchmarked against nine alternatives, including common imputation methods, column-wise deletion with a missingness mask (CW + M), and two MICE variants (MICE O and MICE). All non-MICE imputation methods are fitted using covariates only (no outcome labels). MICE O includes outcome information (Nelson-Aalen estimate and event indicator) during imputation following established recommendations [31, 32], while MICE excludes it. For all non-MICE imputers, the imputation model is fitted on the training set and subsequently applied to the validation and test sets via transform. MICE methods are instead jointly fitted on the training and validation sets; at test time, the learned regression imputation models are used to impute missing values, since MICE’s iterative procedure cannot be directly applied to new observations. MICE methods were averaged over five imputations and five simulation runs. Model performance is evaluated using standard survival metrics:

- **Time-Dependent Concordance Index (**${\textbf {C}}^{{\textbf {td}}}$**)** [33] A discrimination metric assessing the ordering of predicted risks. Higher values (maximum 1) indicate better performance.
- **Integrated Brier Score (IBS)** The Brier score [34] measures the mean squared error between observed outcomes and predicted probabilities, adjusted for censoring. IBS averages this over time, with lower values indicating better performance.
- **Integrated Negative Binomial Log-Likelihood (INBLL)** Measures binary prediction performance using the negative log-likelihood, adjusted for censoring and integrated over time. Lower values indicate better performance.
- **Negative Right-Censored Log-Likelihood** The survival log-likelihood which is a proper scoring rule [35], with lower values indicating better model fit.
- **Integrated Square Error (ISE)** Measures the average discrepancy between observed and predicted risk curves over a time interval.

We place greater emphasis on the negative right-censored log-likelihood and ISE, since the $\text {C}^{\text {td}}$ is not a proper scoring rule, and IBS is only proper when the inverse probability of censoring weights (IPCW) are correctly specified [35].

### Experimental Setup

We split the data into 64% training, 16% validation, and 20% testing. The validation set is used for model selection and early stopping, and the test set for final evaluation. Simulations use 5 random seeds to assess average performance, while real-world EHR data is evaluated via 5-fold cross-validation. In MissCVAE, the ELBO is adjusted by upweighting survival loss and downweighting KL loss to enhance prediction and prevent KL vanishing:

$$\begin{aligned} \text {ELBO}_{\text {modified}}&= \alpha \cdot \text {Survival Loss} \\&\quad + \boldsymbol{\beta } \cdot \text {Reconstruction Loss} \\&\quad + \boldsymbol{\gamma } \cdot \text {KL Loss} \end{aligned}$$

where $\alpha =10$, $\boldsymbol{\beta }={(\beta _M, \beta _X)}$ and $\beta _M=\beta _X=0.1$, $\boldsymbol{\gamma }={(\gamma _M, \gamma _X)}$ and $\gamma _M=\gamma _X=0.1$.

The proposed framework is designed to work with any model, provided all components can be trained end-to-end, enabling joint optimisation across the entire architecture. For our experiments, we demonstrate its application using DeSurv [36], which offers continuous survival curves and incorporates a well-justified competing risks component (full model details are provided in the Supplement Section I.D.). We conduct an ablation study to assess the framework’s performance independent of specific parameter configurations (see Supplementary Table 6). Key analyses include removing the missing embedding as input to the prediction network, adjusting ELBO weights, and varying latent space dimensions both smaller and larger than the baseline. Details are provided in the Supplementary Section V.

### Evaluation Scenarios

We comprehensively assess model performance across multiple scenarios. As $\Delta$-adjustment methods in pattern–mixture models are not directly applicable to our non-imputation framework, we instead assess robustness through missingness shift simulations and distributional shifts across datasets.

- **Baseline.** Models are trained and tested on datasets with the same distribution of missingness, providing a reference across all experiments.
- **Unseen Missingness Patterns.** Models are trained on a subset of missingness patterns (e.g., A, C, D) and evaluated on unseen ones (e.g., B) withheld from training and validation. These unseen patterns are created through structured, non-random removal of cases exhibiting specific configurations of observed variables (e.g., 3 or 4 observed out of 5), reflecting systematic missingness mechanisms.
- **Missingness Shift (Simulation).** Models are trained on datasets with shifted missingness (via Step 4) but identical Steps 1–3. Testing is done on the baseline test set.
- **Distribution Shift (Real World Datasets).** Models are trained on data from London and tested on data from North East England.

## Results

### Simulation

Table 2 and Supplementary Table 7 shows the results from the three simulation scenarios. In our simulations, the variables are designed to be both intrinsically informative and for their absence to carry predictive information about the outcome. Under these conditions, the CW method exhibits the worst performance and incorporating missing indicator (CW + M) leads to modest improvements. The proposed MissCVAE framework and its variant that excludes $\textbf{h}_m$ (labelled as MissCVAE (${\textbf {h}}_x$)) as input to the survival head, achieve performance comparable to the strongest alternative method across all scenarios, according to standard survival metrics ($\text {C}^{\text {td}}$, IBS, INBLL). We note however that these standard metrics are not proper scoring rules [35] which means that the true distribution may be scored worse than incorrect distributions and can lead to inaccurate inferences about performance. Therefore, when using negative right-censored survival log-likelihood, as well as the ground-truth data using the ISE metric, the utility of MissCVAE - which achieved lower ISE throughout - is more clearly demonstrated (this is something to note when considering the real data analysis when the ISE metric is unavailable).

**Table 2:** Comparison of model performance in the simulation studies, reported as mean (standard deviation) over 5 simulations. The best performing benchmark is highlighted in **bold**, with MissCVAE variants that perform comparably or outperform the best benchmark score also highlighted in **bold**

Simulation	Method	C$^{\text {td}}$ $\uparrow$	IBS $\downarrow$	INBLL $\downarrow$	-Loglikelihood $\downarrow$	ISE $\downarrow$
A:	CW	0.699 (0.004)	0.161 (0.001)	0.486 (0.003)	0.255 (0.003)	0.512 (0.004)
Baseline	CW + M	0.721 (0.003)	0.154 (0.002)	0.469 (0.004)	0.239 (0.005)	0.374 (0.004)
	Mean	0.747 (0.004)	0.144 (0.002)	0.444 (0.005)	0.213 (0.006)	0.176 (0.002)
	ICE	0.747 (0.004)	0.144 (0.002)	0.444 (0.006)	0.213 (0.006)	0.175 (0.006)
	RI	0.747 (0.004)	0.144 (0.002)	0.443 (0.006)	0.211 (0.006)	0.167 (0.003)
	MICE O	0.746 (0.004)	0.144 (0.002)	0.444 (0.005)	0.214 (0.005)	0.182 (0.003)
	MICE	0.743 (0.004)	0.146 (0.002)	0.448 (0.004)	0.217 (0.005)	0.206 (0.005)
	-1 Enc.	0.742 (0.004)	0.146 (0.002)	0.449 (0.005)	0.218 (0.006)	0.214 (0.008)
	MIM	**0.749 (0.004)**	**0.143 (0.002)**	**0.441 (0.005)**	**0.210 (0.006)**	**0.158 (0.005)**
	MissCVAE	**0.750 (0.003)**	**0.143 (0.002)**	0.442 (0.005)	0.211 (0.005)	**0.158 (0.005)**
	MissCVAE (${\textbf {h}}_x$)	**0.750 (0.003)**	**0.143 (0.002)**	0.442 (0.004)	0.211 (0.005)	**0.159 (0.004)**
B:	CW	0.677 (0.009)	0.185 (0.003)	0.545 (0.008)	0.234 (0.005)	0.718 (0.023)
Unseen	CW + M	0.694 (0.012)	0.173 (0.004)	0.515 (0.009)	0.205 (0.008)	0.490 (0.024)
Patterns	Mean	0.724 (0.007)	0.160 (0.003)	0.481 (0.007)	0.171 (0.014)	0.254 (0.013)
(Subset)	ICE	0.689 (0.034)	0.185 (0.024)	0.691 (0.247)	0.495 (0.454)	0.730 (0.454)
	RI	0.725 (0.006)	**0.159 (0.002)**	**0.480 (0.005)**	0.170 (0.011)	0.231 (0.015)
	MICE O	0.726 (0.006)	**0.159 (0.001)**	0.481 (0.005)	0.171 (0.009)	**0.222 (0.010)**
	MICE	0.724 (0.007)	0.161 (0.002)	0.486 (0.005)	0.175 (0.006)	0.256 (0.016)
	-1 Enc.	0.716 (0.009)	0.164 (0.003)	0.494 (0.007)	0.184 (0.006)	0.344 (0.020)
	MIM	**0.727 (0.007)**	**0.159 (0.001)**	0.481 (0.004)	**0.169 (0.011)**	0.224 (0.020)
	MissCVAE	**0.728 (0.008)**	**0.158 (0.001)**	**0.477 (0.003)**	**0.168 (0.010)**	**0.196 (0.010)**
	MissCVAE (${\textbf {h}}_x$)	**0.728 (0.007)**	**0.159 (0.001)**	**0.479 (0.003)**	**0.169 (0.011)**	**0.207 (0.013)**
C:	CW	0.699 (0.004)	0.161 (0.001)	0.486 (0.003)	0.255 (0.003)	0.512 (0.004)
Missingness	CW + M	0.716 (0.004)	0.158 (0.002)	0.479 (0.005)	0.248 (0.006)	0.444 (0.013)
Shift	Mean	0.747 (0.003)	**0.144 (0.002)**	0.444 (0.004)	0.213 (0.005)	0.176 (0.004)
	ICE	0.747 (0.004)	**0.144 (0.002)**	0.444 (0.005)	0.213 (0.005)	0.175 (0.006)
	RI	**0.748 (0.003)**	**0.144 (0.002)**	**0.443 (0.005)**	**0.211 (0.006)**	**0.166 (0.005)**
	MICE O	0.746 (0.003)	**0.144 (0.002)**	0.444 (0.005)	0.213 (0.005)	0.178 (0.004)
	MICE	0.744 (0.003)	0.145 (0.002)	0.447 (0.004)	0.216 (0.005)	0.202 (0.006)
	-1 Enc.	0.739 (0.005)	0.148 (0.003)	0.455 (0.006)	0.223 (0.007)	0.251 (0.012)
	MIM	**0.748 (0.004)**	**0.144 (0.002)**	**0.443 (0.005)**	0.212 (0.006)	0.171 (0.009)
	MissCVAE	**0.749 (0.004)**	**0.144 (0.002)**	**0.443 (0.004)**	0.213 (0.006)	**0.163 (0.006)**
	MissCVAE (${\textbf {h}}_x$)	**0.749 (0.004)**	**0.144 (0.002)**	**0.443 (0.005)**	0.212 (0.006)	**0.165 (0.010)**

In Simulation A, the MIM method which flexibly incorporates missingness information scores best amongst all metrics, particularly in log-likelihood and ISE, against existing imputation methods. However, MissCVAE achieves competitive performance, with no statistically significant difference detected compared to baselines, especially when training is weighted towards predictive performance ($\alpha =10$). In Simulations B, when models are tested on missingness patterns that were unseen during training, MICE-O was competitive with MIM but, on log-likelihood and ISE, MissCVAE showed better performance. However, when moving to Simulation C, we observe that methods that leverage informative missingness exhibit prediction degradation under missingness shift. For example, in terms of ISE, MIM degrades from 0.158 to 0.171, whereas our proposed method demonstrates greater robustness, with a smaller drop from 0.158 to 0.163. Despite this, it still outperforms all other benchmarks, with the next-best method RI achieving an ISE of 0.166. Overall, while the performance of existing missingness handling is sensitive to the actual mechanisms at play, the behaviour of MissCVAE appears consistent across the different scenarios.

These results align with our findings on how varying missingness patterns affect predictions for the same individual (see Supplementary Section V). By masking input features and passing incomplete data through trained models, we observe that methods explicitly modelling missingness exhibit the greatest prediction variability, reflecting their reliance on the missingness signal. Among them, our proposed method shows the smallest variation, indicating greater robustness and a balance between leveraging missingness and avoiding overfitting (Supplementary Fig. 2). In contrast, imputation-based methods appear more stable but can be unreliable in extreme cases, where imputations deviate significantly from true values (Supplementary Table 14). This can lead to underestimation of risk in severely ill patients or overestimation in very healthy ones when there are high levels of missingness. Despite not explicitly modelling missingness, these methods can still implicitly encode its structure, resulting in prediction curves similar to models that do.

In our ablation studies, we find that assigning a higher weight to the survival prediction term $\alpha$ in the ELBO is essential for maintaining strong performance. Across all simulation scenarios, removing the missingness representation ${\textbf {h}}_m$ from the prediction head consistently leads to degraded performance, consistent with the presence of informative missingness in the simulated data. When using a higher $\alpha$ (or lower $\beta$ or $\gamma$), most parameterisations perform at least as well as imputation-based benchmarks in Simulation A (though not as well as MIM), outperform the best benchmark in Simulation B and surpass MIM in Simulation C in terms of ISE.

### Real-World Primary Care Data

We next considered the analysis of the CPRD-derived data sets and results are shown in Tables 3, 4, and 5, as well as Supplementary Table 11. On the CKD dataset, the CW method performed worst across all metrics, and its performance deteriorated further when the missingness mask was included (CW+M), indicating potential overfitting to missingness patterns. Among existing approaches, ICE, RI, and MIM performed well under unseen missingness patterns, while MICE O and the -1 encoding method were also competitive under distribution shift. However, both variants of MissCVAE achieved competitive performance under $\text {C}^{\text {td}}$, IBS, and INBLL, and obtained improved likelihood scores in both scenarios, demonstrating an ability to exploit missingness structure while remaining robust to overfitting. Consistent findings were observed in the baseline analysis in the Supplementary Materials.

**Table 3:** Comparison of model performance in the CKD dataset, reported as mean (standard deviation) over 5 folds. The best performing benchmark is highlighted in **bold**, with MissCVAE variants that perform comparably or outperform the best benchmark score also highlighted in **bold**

Experiment	Method	C$^{\text {td}}$ $\uparrow$	IBS $\downarrow$	INBLL $\downarrow$	-Likelihood $\downarrow$
Unseen Patterns	CW	0.610 (0.005)	0.185 (0.003)	0.545 (0.007)	0.323 (0.006)
	CW + M	0.611 (0.009)	0.185 (0.002)	0.544 (0.005)	0.326 (0.006)
	Mean	0.775 (0.006)	0.144 (0.002)	0.445 (0.008)	0.217 (0.010)
	ICE	0.780 (0.007)	**0.143 (0.003)**	0.442 (0.009)	0.211 (0.011)
	RI	0.780 (0.006)	**0.143 (0.003)**	0.441 (0.008)	**0.210 (0.012)**
	MICE O	0.778 (0.007)	0.144 (0.003)	0.442 (0.007)	0.213 (0.012)
	MICE	0.776 (0.007)	0.145 (0.002)	0.446 (0.005)	0.216 (0.012)
	-1 Enc.	0.758 (0.009)	0.151 (0.003)	0.463 (0.010)	0.234 (0.017)
	MIM	**0.781 (0.005)**	**0.143 (0.003)**	**0.440 (0.010)**	0.211 (0.011)
	MissCVAE	**0.781 (0.006)**	**0.142 (0.002)**	**0.438 (0.007)**	**0.209 (0.014)**
	MissCVAE (${\textbf {h}}_x$)	**0.781 (0.008)**	**0.142 (0.002)**	**0.439 (0.006)**	**0.206 (0.014)**
Distribution Shift	CW	0.630 (0.002)	0.173 (0.001)	0.513 (0.002)	0.346 (0.003)
	CW + M	0.636 (0.003)	0.173 (0.001)	0.514 (0.003)	0.365 (0.008)
	Mean	0.758 (0.001)	0.145 (0.001)	0.445 (0.001)	0.285 (0.002)
	ICE	**0.762 (0.001)**	**0.144 (0.001)**	0.443 (0.002)	0.282 (0.001)
	RI	**0.762 (0.001)**	**0.762 (0.001)**	0.443 (0.000)	0.282 (0.001)
	MICE O	0.761 (0.001)	**0.144 (0.000)**	**0.441 (0.001)**	**0.280 (0.001)**
	MICE	0.758 (0.001)	0.145 (0.001)	0.443 (0.001)	0.283 (0.001)
	-1 Enc.	0.755 (0.002)	0.145 (0.001)	0.444 (0.002)	0.281 (0.005)
	MIM	**0.762 (0.001)**	**0.144 (0.000)**	0.444 (0.001)	0.284 (0.001)
	MissCVAE	0.761 (0.003)	**0.144 (0.001)**	**0.441 (0.003)**	**0.268 (0.013)**
	MissCVAE (${\textbf {h}}_x$)	0.761 (0.001)	**0.144 (0.000)**	**0.441 (0.001)**	**0.254 (0.005)**

**Table 4:** Comparison of model performance in the CVD dataset, reported as mean (standard deviation) over 5 folds. The best performing benchmark is highlighted in **bold**, with MissCVAE variants that perform comparably or outperform the best benchmark score also highlighted in **bold**

Experiment	Method	C$^{\text {td}}$ $\uparrow$	IBS $\downarrow$	INBLL $\downarrow$	-Likelihood $\downarrow$
Unseen Patterns	CW	0.555 (0.019)	0.111 (0.006)	0.363 (0.015)	0.230 (0.010)
	CW + M	0.557 (0.015)	**0.110 (0.006)**	**0.362 (0.016)**	**0.229 (0.011)**
	Mean	0.575 (0.018)	0.112 (0.007)	0.366 (0.017)	**0.229 (0.012)**
	ICE	**0.577 (0.019)**	0.112 (0.007)	0.366 (0.017)	**0.229 (0.011)**
	RI	0.575 (0.019)	0.112 (0.006)	0.366 (0.017)	**0.229 (0.012)**
	MICE O	0.574 (0.019)	0.111 (0.006)	0.365 (0.017)	**0.229 (0.011)**
	MICE	0.573 (0.018)	0.111 (0.006)	0.366 (0.016)	0.230 (0.011)
	-1 Enc.	0.560 (0.014)	0.111 (0.006)	0.365 (0.016)	**0.229 (0.011)**
	MIM	0.576 (0.008)	0.111 (0.006)	0.365 (0.017)	**0.229 (0.012)**
	MissCVAE	0.576 (0.016)	0.111 (0.006)	0.365 (0.015)	0.231 (0.010)
	MissCVAE (${\textbf {h}}_x$)	**0.579 (0.017)**	0.111 (0.006)	0.364 (0.015)	0.230 (0.011)
Distribution Shift	CW	0.563 (0.005)	**0.126 (0.000)**	0.402 (0.001)	0.242 (0.000)
	CW + M	**0.572 (0.003)**	**0.126 (0.000)**	**0.401 (0.001)**	**0.241 (0.001)**
	Mean	0.565 (0.004)	0.127 (0.000)	0.406 (0.001)	0.242 (0.001)
	ICE	0.567 (0.003)	0.127 (0.000)	0.405 (0.001)	0.242 (0.000)
	RI	0.567 (0.004)	0.127 (0.000)	0.406 (0.001)	0.242 (0.000)
	MICE O	0.566 (0.002)	0.127 (0.000)	0.404 (0.001)	0.242 (0.000)
	MICE	0.566 (0.002)	0.127 (0.000)	0.403 (0.001)	0.242 (0.000)
	-1 Enc.	0.572 (0.004)	0.126 (0.000)	**0.401 (0.001)**	**0.241 (0.001)**
	MIM	**0.572 (0.004)**	0.127 (0.001)	0.404 (0.002)	**0.241 (0.001)**
	MissCVAE	**0.574 (0.004)**	**0.126 (0.000)**	0.402 (0.001)	**0.241 (0.001)**
	MissCVAE (${\textbf {h}}_x$)	**0.574 (0.004)**	0.127 (0.000)	0.403 (0.001)	**0.241 (0.000)**

**Table 5:** Model performance in the *All-Cause Mortality dataset*, reported as mean (standard deviation) over 5 folds. The best performing benchmark is highlighted in **bold**, with all MissCVAE variants that perform comparably or outperform the best benchmark score also highlighted in **bold**

Experiment	Method	C$^{\text {td}}$ $\uparrow$	IBS $\downarrow$	INBLL $\downarrow$	-Likelihood $\downarrow$
Unseen Patterns	CW	0.689 (0.008)	0.096 (0.002)	0.314 (0.005)	**0.406 (0.008)**
	CW + M	0.688 (0.009)	0.097 (0.002)	0.318 (0.006)	0.423 (0.010)
	Mean	0.692 (0.009)	0.096 (0.001)	0.316 (0.005)	0.421 (0.013)
	ICE	**0.698 (0.008)**	**0.095 (0.002)**	**0.312 (0.005)**	0.412 (0.011)
	RI	0.693 (0.007)	**0.095 (0.002)**	0.314 (0.005)	0.416 (0.011)
	MICE O	0.687 (0.007)	0.097 (0.002)	0.318 (0.004)	0.424 (0.005)
	MICE	0.694 (0.009)	**0.095 (0.002)**	0.313 (0.004)	0.412 (0.008)
	-1 Enc.	0.691 (0.008)	**0.095 (0.002)**	0.314 (0.005)	0.415 (0.009)
	MIM	0.696 (0.007)	0.096 (0.001)	0.316 (0.004)	0.422 (0.006)
	MissCVAE	**0.699 (0.008)**	**0.094 (0.002)**	**0.311 (0.005)**	0.410 (0.011)
	MissCVAE (${\textbf {h}}_x$)	**0.700 (0.009)**	**0.094 (0.002)**	**0.311 (0.004)**	0.408 (0.011)
Distribution Shift	CW	0.695 (0.001)	0.102 (0.000)	0.327 (0.000)	**0.460 (0.001)**
	CW + M	0.693 (0.001)	0.102 (0.000)	0.327 (0.000)	0.466 (0.001)
	Mean	0.705 (0.003)	**0.100 (0.000)**	0.322 (0.001)	0.462 (0.002)
	ICE	0.702 (0.003)	0.101 (0.000)	0.323 (0.001)	**0.460 (0.002)**
	RI	0.702 (0.003)	0.101 (0.000)	0.323 (0.001)	0.461 (0.001)
	MICE O	0.696 (0.002)	0.102 (0.001)	0.328 (0.002)	0.475 (0.003)
	MICE	0.699 (0.002)	0.101 (0.000)	0.325 (0.001)	0.468 (0.001)
	-1 Enc.	0.698 (0.002)	0.101 (0.000)	0.324 (0.001)	0.463 (0.002)
	MIM	**0.706 (0.003)**	**0.100 (0.001)**	**0.321 (0.002)**	0.466 (0.003)
	MissCVAE	0.700 (0.001)	0.101 (0.000)	0.323 (0.001)	**0.460 (0.001)**
	MissCVAE (${\textbf {h}}_x$)	0.701 (0.002)	0.101 (0.000)	0.323 (0.001)	**0.460 (0.001)**

In the CVD experiments, all models achieved similarly low concordance scores (circa 0.57), and even methods using complete features only performed comparably with other approaches. This suggests that the predictors in this dataset contain limited intrinsic signal, and consequently the choice of missingness strategy has little influence on predictive performance. This example reinforces that the effectiveness of missingness-handling approaches is ultimately constrained by the predictive information available in the underlying features.

In the ACM study, CW attained the strongest survival likelihood despite performing worse on the standard survival metrics. This dataset contains a substantially higher proportion of missingness than the previous two, and Fig. 2 suggests that the missingness pattern itself carries predictive signal. In this case, simple deletion may outperform more complex handling because the remaining observed values can be more informative than potentially noisy imputations. The CW+M variant performed notably worse, indicating that directly incorporating the missingness mask may lead to overfitting rather than effectively exploiting this signal. In contrast, MissCVAE remained competitive across all metrics in both scenarios, demonstrating robustness to high missingness and the ability to leverage its structure without overfitting.

In our ablation studies (Supplementary Tables 12 and 13), we observe that nearly all parameterisations except those with a lower $\alpha$ weight or smaller latent dimensionality, outperform the best benchmark across the IBS, INBLL, and negative log-likelihood metrics in the CKD experiments. In the CVD experiments, the ablation that uses only $\textbf{h}_x$ for prediction (i.e., excluding $\textbf{h}_m$) performs best under the unseen missingness patterns. Our approach does not outperform other methods in the CVD setting. This slightly lower performance is likely attributable to stronger regularisation and reduced flexibility in the proposed framework, which may limit its ability to capture weak predictive signals in this dataset.

**Fig. 6:**
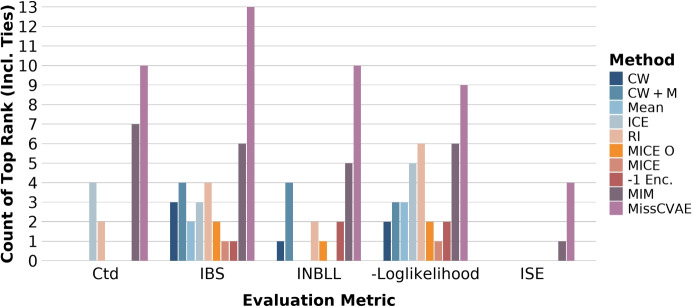
Total count of top rank (including ties) across simulation and real data analysis scenarios

Overall, Fig. 6 shows how often each method ranked first (including ties) across all simulation and real-world studies, with MissCVAE most frequently achieving top performance. This evidence supports MissCVAE as a reliable and broadly effective approach for handling missingness in survival prediction.

## Conclusion

In this article, we emphasise the importance of deploying predictive models that can effectively handle missing data in real-world predictions. Poor handling of missingness can compromise model accuracy, leading to suboptimal or even harmful clinical decisions. It is crucial that, in contrast to inference methods, prediction models trained and validated on historical data must be directly applicable to new predictions without modification.

We propose an imputation-free deep learning framework as a practical alternative to handling missing data in survival risk prediction. Unlike traditional approaches that rely on imputation, our framework directly models missingness patterns, ensuring a more seamless and reliable integration of incomplete data. The framework is designed for end-to-end compatibility with any differentiable neural network used for survival prediction.

A key limitation of real-world survival analysis is the absence of ground-truth survival probabilities. Unlike simulations where true risk functions are known, real-world data only provide observed event or censoring times, which are step-function realisations of an underlying stochastic process. Predicted survival curves, on the other hand, approximate the full probability distribution, and this mismatch makes it difficult to directly assess individual level predictions. As a result, evaluation relies on metrics such as $\text {C}^{\text {td}}$, IBS, or negative log-likelihood, which cannot assess full fidelity to the true survival function. We note that only the likelihood-based metric is a proper scoring rule, while the C-index is non-proper and the properness of the IBS depends on correctly specified censoring weights.

Future work will extend the framework to additional feature types and modalities. Another direction is to learn shared structure across related patterns of missingness, for example through hierarchical regularisation or gated mechanisms, to better capture commonalities without overfitting. Finally, incorporating attention or transformer based architectures into MissCVAE may help capture time dependent patterns of missingness and improve modelling of long range dependencies in EHR data.

### Supplementary information

Supplementary material is available at Journal of Healthcare Informatics Research online and is structured as follows: Section I introduces the technical background in survival analysis; Section II details the proposed model mathematical derivations; Section III presents additional simulation details; Section IV provides further information on the real-world data sets; and Section V outlines experimental implementation settings and additional results.

## Supplementary Information

Below is the link to the electronic supplementary material.

Supplementary file 1 (pdf 2635 KB)

## Data Availability

This study is based on data from the Clinical Practice Research Datalink (CPRD). Data from CPRD was obtained under an approved protocol with Study Reference ID (22_001903). The data cannot be shared publicly due to licensing restrictions. Access to CPRD data is available only via application to the Medicines and Healthcare products Regulatory Agency (MHRA) and requires relevant approvals. Researchers can apply for data access through the CPRD website (www.cprd.com) in accordance with the CPRD Data Governance policies.

## References

[1] Haneuse S, Daniels M (2016) A general framework for considering selection bias in EHR-based studies: What data are observed and why? EGEMS (Wash, DC) 4(1):1203. 10.13063/2327-9214.1203 10.13063/2327-9214.1203 PMC5013936 27668265

[2] Weiskopf NG, Rusanov A, Weng C (2013) Sick patients have more data: the non-random completeness of electronic health records. AMIA Annual Symposium proceedings AMIA Symposium 2013:1472–1477 24551421 PMC3900159

[3] Rusanov A, Weiskopf NG, Wang S, Weng C (2014) Hidden in plain sight: bias towards sick patients when sampling patients with sufficient electronic health record data for research. BMC Med Inform Decis Mak 14(1):51. 10.1186/1472-6947-14-51 24916006 10.1186/1472-6947-14-51 PMC4062889

[4] Getzen E, Ungar L, Mowery D, Jiang X, Long Q (2023) Mining for equitable health: Assessing the impact of missing data in electronic health records. J Biomed Inform 139:104269. 10.1016/j.jbi.2022.104269 36621750 10.1016/j.jbi.2022.104269 PMC10391553

[5] Sisk R, Lin L, Sperrin M, Barrett JK, Tom B, Diaz-Ordaz K et al (2020) Informative presence and observation in routine health data: A review of methodology for clinical risk prediction. J Am Med Inf Assoc 28(1):155–166. https://doi.org/10.1093/jamia/ocaa242. https://academic.oup.com/jamia/article-pdf/28/1/155/35885573/ocaa242.pdf 10.1093/jamia/ocaa242 PMC7810439 33164082

[6] Agniel D, Kohane IS, Weber GM (2018) Biases in electronic health record data due to processes within the healthcare system: retrospective observational study. BMJ 361. https://doi.org/10.1136/bmj.k1479, https://www.bmj.com/content/361/bmj.k1479.full.pdf 10.1136/bmj.k1479 PMC5925441 29712648

[7] Nijman SWJ, Leeuwenberg AM, Beekers I, Verkouter I, Jacobs JJL, Bots ML et al (2022) Missing data is poorly handled and reported in prediction model studies using machine learning: a literature review. J Clin Epidemiol 142:218–229. 10.1016/j.jclinepi.2021.11.023 34798287 10.1016/j.jclinepi.2021.11.023

[8] Sperrin M, Martin GP, Sisk R, Peek N (2020) Missing data should be handled differently for prediction than for description or causal explanation. J Clin Epidemiol 125:183–187. 10.1016/j.jclinepi.2020.03.028 32540389 10.1016/j.jclinepi.2020.03.028

[9] Lin JH, Haug PJ (2008) Exploiting missing clinical data in Bayesian network modeling for predicting medical problems. J Biomed Inform 41(1):1–14. 10.1016/j.jbi.2007.06.001 17625974 10.1016/j.jbi.2007.06.001

[10] Hoogland J, van Barreveld M, Debray TPA, Reitsma JB, Verstraelen TE, Dijkgraaf MGW et al (2020) Handling missing predictor values when validating and applying a prediction model to new patients. Stat Med 39(25):3591–3607. 10.1002/sim.8682, https://onlinelibrary.wiley.com/doi/pdf/10.1002/sim.8682 10.1002/sim.8682 PMC7586995 32687233

[11] Rubin DB (1976) Inference and missing data. Biometrika 63(3):581–592. https://doi.org/10.1093/biomet/63.3.581, https://academic.oup.com/biomet/article-pdf/63/3/581/756166/63-3-581.pdf

[12] Hippisley-Cox J, Coupland C, Brindle P (2017) Development and validation of QRISK3 risk prediction algorithms to estimate future risk of cardiovascular disease: prospective cohort study. BMJ 357. https://doi.org/10.1136/bmj.j2099, https://www.bmj.com/content/357/bmj.j2099.full.pdf 10.1136/bmj.j2099 PMC5441081 28536104

[13] Fletcher Mercaldo S, Blume JD (2018) Missing data and prediction: the pattern submodel. Biostatistics 21(2):236–252. https://doi.org/10.1093/biostatistics/kxy040. https://academic.oup.com/biostatistics/article-pdf/21/2/236/36208952/kxy040.pdf 10.1093/biostatistics/kxy040 PMC7868046 30203058

[14] Stempfle L, Panahi A, Johansson FD (2023) Sharing Pattern Submodels for Prediction with Missing Values. Proceedings of the AAAI Conference on Artificial Intelligence 37(8):9882–9890. 10.1609/aaai.v37i8.26179

[15] Buck SF (1960) A Method of Estimation of Missing Values in Multivariate Data Suitable for Use with an Electronic Computer. J Roy Stat Soc: Ser B (Methodol) 22(2):302–306. 10.1111/j.2517-6161.1960.tb00375.x, https://rss.onlinelibrary.wiley.com/doi/pdf/10.1111/j.2517-6161.1960.tb00375.x

[16] Rubin DB (1987) Multiple Imputation for Nonresponse in Surveys. Wiley

[17] White IR, Royston P, Wood AM (2011) Multiple imputation using chained equations: Issues and guidance for practice. Stat Med 30(4):377–399. 10.1002/sim.4067, https://onlinelibrary.wiley.com/doi/pdf/10.1002/sim.4067 10.1002/sim.4067 21225900

[18] Janssen KJM, Vergouwe Y, Donders ART, Harrell J Frank E, Chen Q, Grobbee DE et al (2008) Dealing with Missing Predictor Values When Applying Clinical Prediction Models. Clinical Chemistry 55(5):994–1001. https://doi.org/10.1373/clinchem.2008.115345, https://academic.oup.com/clinchem/article-pdf/55/5/994/32673661/clinchem0994.pdf 10.1373/clinchem.2008.115345 19282357

[19] van Buuren S, Groothuis-Oudshoorn K (2011) mice: Multivariate Imputation by Chained Equations in R. J Stat Softw 45(3):1–67. 10.18637/jss.v045.i03

[20] Van Ness M, Bosschieter TM, Halpin-Gregorio R, Udell M (2023) The Missing Indicator Method: From Low to High Dimensions. In: Proceedings of the 29th ACM SIGKDD Conference on Knowledge Discovery and Data Mining. KDD ’23. New York, NY, USA: Association for Computing Machinery, pp 5004–5015. Available from: 10.1145/3580305.3599911

[21] Li J, Wang M, Steinbach MS, Kumar V, Simon GJ (2018) Don’t Do Imputation: Dealing with Informative Missing Values in EHR Data Analysis. In: 2018 IEEE International Conference on Big Knowledge (ICBK), pp 415–422

[22] Kingma DP, Welling M (2019) Auto-Encoding Variational Bayes. CoRR arXiv:1312.6114

[23] Ipsen NB, Mattei PA, Frellsen J (2020) How to deal with missing data in supervised deep learning? In: International conference on learning representations. Available from: https://api.semanticscholar.org/CorpusID:221508842

[24] Mitra R, McGough SF, Chakraborti T, Holmes C, Copping R, Hagenbuch N et al (2023) Learning from data with structured missingness. Nature Machine Intelligence 5(1):13–23. 10.1038/s42256-022-00596-z

[25] Tan ALM, Getzen EJ, Hutch MR, Strasser ZH, Gutiérrez-Sacristán A, Le TT et al (2023) Informative missingness: What can we learn from patterns in missing laboratory data in the electronic health record? J Biomed Inform 139:104306. 10.1016/j.jbi.2023.104306 36738870 10.1016/j.jbi.2023.104306 PMC10849195

[26] Herrett E, Gallagher AM, Bhaskaran K, Forbes H, Mathur R, van Staa T et al (2015) Data Resource Profile: Clinical Practice Research Datalink (CPRD). Int J Epidemiol 44(3):827–836. 10.1093/ije/dyv098 10.1093/ije/dyv098 PMC4521131 26050254

[27] OPTIMAL (2021) OPTIMising therapies, disease trajectories, and AI assisted clinical management for patients Living with complex multimorbidity (OPTIMAL study). Available from: https://fundingawards.nihr.ac.uk/award/NIHR202632

[28] Gokhale KM, Chandan JS, Toulis K, Gkoutos G, Tino P, Nirantharakumar K (2021) Data extraction for epidemiological research (DExtER): a novel tool for automated clinical epidemiology studies. Eur J Epidemiol 36(2):165–178. 10.1007/s10654-020-00677-6 32856160 10.1007/s10654-020-00677-6 PMC7987616

[29] Gokhale KM, Chandan JS, Sainsbury C, Tino P, Tahrani A, Toulis K et al (2024) Using Repeated Measurements to Predict Cardiovascular Risk in Patients With Type 2 Diabetes Mellitus. Am J Cardiol 210:133–142. 10.1016/j.amjcard.2023.10.008 38682712 10.1016/j.amjcard.2023.10.008

[30] Dziopa K, Asselbergs FW, Gratton J, Chaturvedi N, Schmidt AF (2022) Cardiovascular risk prediction in type 2 diabetes: a comparison of 22 risk scores in primary care settings. Diabetologia 65(4):644–656. 10.1007/s00125-021-05640-y 35032176 10.1007/s00125-021-05640-y PMC8894164

[31] Sterne JAC, White IR, Carlin JB, Spratt M, Royston P, Kenward MG et al (2009) Multiple imputation for missing data in epidemiological and clinical research: potential and pitfalls. BMJ 338. https://doi.org/10.1136/bmj.b2393, https://www.bmj.com/content 10.1136/bmj.b2393 PMC2714692 19564179

[32] White IR, Royston P (2009) Imputing missing covariate values for the Cox model. Stat Med 28(15):1982–1998. 10.1002/sim.3618, https://onlinelibrary.wiley.com/doi/pdf/10.1002/sim.3618 10.1002/sim.3618 PMC2998703 19452569

[33] Antolini L, Boracchi P, Biganzoli E (2005) A Time-Dependent Discrimination Index for Survival Data. Stat Med 24(24):3927–3944. 10.1002/sim.2427 16320281 10.1002/sim.2427

[34] Brier GW (1950) Verification of forecasts expressed in terms of probability. Mon Weather Rev 78(1):1–3. 10.1175/1520-0493(1950)078<;0001:VOFEIT>;2.0.CO;2

[35] Rindt D, Hu R, Steinsaltz D, Sejdinovic D (2022) Survival regression with proper scoring rules and monotonic neural networks. In: International conference on artificial intelligence and statistics. PMLR pp 1190–1205

[36] Danks D, Yau C (2022) Derivative-Based Neural Modelling of Cumulative Distribution Functions for Survival Analysis. In: Camps-Valls G, Ruiz FJR, Valera I (ed). Proceedings of The 25th International Conference on Artificial Intelligence and Statistics. vol 151 of Proceedings of Machine Learning Research. PMLR pp 7240–7256. Available from: https://proceedings.mlr.press/v151/danks22a.html

