# Supplementary material for: Imputation Free Deep Survival Prediction with Conditional Variational Autoencoders
Source: J Healthc Inform Res. 2026 Apr 10;10(2):275–98. doi: 10.1007/s41666-026-00234-y (PMC13156366; doi:10.1007/s41666-026-00234-y)
Supplement: Supplementary file 1 — (pdf 2635 KB) [file 41666_2026_234_MOESM1_ESM.pdf]

Supplementary Material

Imputation Free Deep Survival Prediction with Conditional Variational Autoencoders

Natalia Hong[1, 2, 3], Aditya Acharya[4], Krishna Gokhale[4], Jenny Cooper[4], Charles Gadd[1], Francesca Crowe[4], Krishnarajah Nirantharakumar[4,5], and Christopher Yau*[1, 2]

[1]Nuffield Department of Women's & Reproductive Health, University of Oxford, Oxford, United Kingdom
[2]Health Data Research UK, London, United Kingdom
[3]The Alan Turing Institute, London, United Kingdom
[4]Department of Applied Health Sciences, University of Birmingham, Birmingham, United Kingdom
[5]Department of Population Health Sciences, Kings College London, London, United Kingdom

I Background

A Taxonomy of Missing Data Mechanisms

Rubin classifies missing data into three types: Missing Completely at Random (MCAR), Missing at Random (MAR), and Missing Not at Random (MNAR).[1] These mechanisms describe how missingness relates to observed and unobserved values. Let $\mathbf{m}_i$ denote whether data point $\mathbf{x}_i$ is observed ($\mathbf{m}_i = 1$) or missing ($\mathbf{m}_i = 0$).

- **MCAR**: Missingness occurs purely by chance, independent of both observed and unobserved data (e.g., data loss due to a power outage).

$$P(\mathbf{m}_i = 0 \mid \mathbf{x}_{\text{obs}}, \mathbf{x}_{\text{mis}}) = P(\mathbf{m}_i = 0)$$

- **MAR**: Missingness depends only on observed data but not on the missing values themselves (e.g., younger patients being less likely to have cholesterol levels recorded).

$$P(\mathbf{m}_i = 0 \mid \mathbf{x}_{\text{obs}}, \mathbf{x}_{\text{mis}}) = P(\mathbf{m}_i = 0 \mid \mathbf{x}_{\text{obs}})$$

- **MNAR**: Missingness is directly related to unobserved values (e.g., heavy drinkers being less likely to report alcohol consumption).

$$P(\mathbf{m}_i = 0 \mid \mathbf{x}_{\text{obs}}, \mathbf{x}_{\text{mis}}) = P(\mathbf{m}_i = 0 \mid \mathbf{x}_{\text{mis}})$$

*Corresponding author: christopher.yau@wrh.ox.ac.uk

B Kaplan-Meier Estimator

The Kaplan-Meier estimator is a non-parametric method for estimating the survival function $S(t) = P(T > t)$ from censored time-to-event data.[2] Given n individuals with observed survival times $t_1, t_2, \ldots, t_n$, some of which may be censored, the estimator is defined as:

$$\hat{S}(t) = \prod_{t_i \le t} \left(1 - \frac{d_i}{n_i}\right),$$

where t_i are observed event times, d_i is the number of events at t_i, and n_i is the number at risk just before t_i. This estimator therefore accounts for censoring by only considering individuals at risk at each event time. The resulting survival curve is a step function that decreases at observed failure times. While this makes no assumptions about the underlying distribution, its accuracy declines over time as fewer individuals remain at risk, increasing variance in the estimate.

C Survival Metrics

C.1 Time-Dependent Concordance

The Time-Dependent Concordance Index (Ctd) as defined by Antolini is a discriminative index used to assess the ordering of predicted risks of pairs of individuals based on the earlier event time of each pair.[3] This assumes that an individual i experiencing an event at time t_i should be assigned a higher cumulative risk at time t_i than another individual j who has not yet experienced the event at that time. Formally, this is defined as

$$\text{Ctd} = \mathbb{P}\left(\hat{F}\left(t_i|\mathrm{x}_i\right) > \hat{F}\left(t_i|\mathrm{x}_j\right) \middle| t_i < t_j\right).$$

In practice, this quantity is estimated by calculating the proportion of pairs in concordance

$$\text{Ctd} \approx \frac{\sum_{i \neq j} \mathbb{1}\left(t_i < t_j\right) \cdot \mathbb{1}\left(\hat{F}\left(t_i|\mathbf{x}_i\right) > \hat{F}\left(t_i|\mathbf{x}_j\right)\right)}{\sum_{i \neq j} \mathbb{1}\left(t_i < t_j\right)}.$$

Higher values of Ctd indicate better performance up to the maximum value of 1, but sizeable censoring can lead to an optimistic performance.[4]

C.2 Integrated Brier Score

The Brier score (BS) is a measure of both discrimination and calibration performance as it quantifies the mean squared error between observed event indicators and predicted probabilities at a fixed time point.[5] Censoring is accounted for using inverse probability of censoring weighting.[6,7] The Brier score at time u is given by:

$$\text{BS}(u) = \frac{1}{N}\sum_{i=1}^{N}\left[\frac{\left(1 - \hat{F}(u|\mathbf{x}_i)\right)^2 \mathbb{1}\{t_i \le u\}}{\hat{G}(t_i)} + \frac{\hat{F}\left(u|\mathbf{x}_i\right)^2 \mathbb{1}\{t_i > u\}}{\hat{G}(u)}\right],$$

where $\hat{G}(u)$ is the Kaplan-Meier estimate (Section B) of the censoring distribution. The Integrated Brier Score (IBS) is obtained by averaging the Brier score over a time interval:

$$\text{IBS} = \frac{1}{t_2 - t_1}\int_{t_1}^{t_2} \text{BS}(u)\,\mathrm{d}u.$$

Lower IBS values indicate better overall predictive performance.

C.3 Integrated Negative Binomial Log-Likelihood

The Negative Binomial Log-Likelihood (NBLL) computes the binomial log-likelihood, negated for consistency with loss minimisation. Unlike discrimination-based measures, which assess only ranking, NBLL evaluates the overall likelihood of observed event times under the estimated risk function, making it sensitive to both calibration and discrimination. Similar to the Brier score, IPCW is applied to account for censoring.[6,8] The IPCW-adjusted NBLL at time u is given by:

$$\text{NBLL}(u) = -\frac{1}{N}\sum_{i=1}^{N}\left[\frac{\log \hat{F}(u \mid \mathbf{x}_i)\,\mathbb{1}\{t_i \leq u\}}{\hat{G}(t_i)} + \frac{\log\left(1-\hat{F}(u \mid \mathbf{x}_i)\right)\mathbb{1}\{t_i > u\}}{\hat{G}(u)}\right].$$

To summarise performance over time, the Integrated Negative Binomial Log-Likelihood (INBLL) is computed by averaging the NBLL over a time interval:

$$\text{INBLL} = \frac{1}{t_2 - t_1}\int_{t_1}^{t_2} \text{NBLL}(u)\,\mathrm{d}u.$$

Lower INBLL values indicate better predictive performance, as they reflect higher likelihood of observed events under the estimated model.

C.4 Integrated Square Error (ISE)

Simulation studies provide a controlled setting to evaluate the calibration of absolute risk predictions, as the true risk functions are known. The Integrated Squared Error (ISE) quantifies the discrepancy between the estimated and true cumulative risk functions over time by computing the squared difference at each time point and integrating over the time interval. Averaging across N observations gives:

$$\text{ISE} = \frac{1}{N}\sum_{i=1}^{N}\int_{t_1}^{t_2}\left(F(u|\mathbf{x}_i) - \hat{F}(u|\mathbf{x}_i)\right)^2 \mathrm{d}u.$$

This metric provides a global assessment of the accuracy of the estimated risk, with larger values indicating greater deviation from the true risk over time.

D DeSurv

We illustrate our framework with the deep survival model DeSurv in our experiments.[9] DeSurv provides continuous absolute risk curve predictions, where absolute risk curves correspond to cumulative distribution functions (CDFs), $F(t, \mathbf{x})$. Since a valid CDF is a monotonic function bounded within $[0, 1]$, its derivative is always non-negative.

Leveraging this property, DeSurv models the gradient of an underlying function $u(t, \mathbf{x})$, ensuring monotonicity by constraining the neural network output to be positive via a final softplus activation. The gradient function is then integrated using Gauss-Legendre quadrature, yielding a strictly increasing function $u(t, \mathbf{x})$. To obtain a valid CDF, the underlying function is rescaled from $[0, \infty]$ to $[0, 1]$ using a hyperbolic tangent activation:

$$\frac{\mathrm{d}u}{\mathrm{d}t} = \text{softplus}(NN(t, \mathbf{x}; \theta))$$
$$F(t, \mathbf{x}) = \tanh(u(t, \mathbf{x}))$$

The model is trained using a right-censored survival likelihood as the objective function.

II Proposed Model

A ELBO Derivation

The VAE-cVAE framework in MissCVAE is trained by maximising the marginal likelihood of the observed observations under the model. The Evidence Lower Bound (ELBO) is derived by expanding the log marginal likelihood and using Jensen's Inequality:

$$
\begin{aligned}
\log p_\theta(\mathbf{x}^{\text{obs}}, \mathbf{m}, \mathbf{t}) &= \log \int_{\mathbf{h}_x, \mathbf{h}_m} p_\theta(\mathbf{x}^{\text{obs}}, \mathbf{m}, \mathbf{t}, \mathbf{h}_x, \mathbf{h}_m)\ \mathrm{d}\mathbf{h}_x \mathrm{d}\mathbf{h}_m \\
&= \log \int_{\mathbf{h}_x, \mathbf{h}_m} p_\theta(\mathbf{x}^{\text{obs}}, \mathbf{m}, \mathbf{t}, \mathbf{h}_x, \mathbf{h}_m) \frac{q_\phi(\mathbf{h}_x, \mathbf{h}_m | \mathbf{x}^{\text{obs}}, \mathbf{m})}{q_\phi(\mathbf{h}_x, \mathbf{h}_m | \mathbf{x}^{\text{obs}}, \mathbf{m})}\ \mathrm{d}\mathbf{h}_x \mathrm{d}\mathbf{h}_m \\
&= \log \left(\mathbb{E}_{\mathbf{h}_x, \mathbf{h}_m \sim q_\phi} \left[\frac{p_\theta(\mathbf{x}^{\text{obs}}, \mathbf{m}, \mathbf{t}, \mathbf{h}_x, \mathbf{h}_m)}{q_\phi(\mathbf{h}_x, \mathbf{h}_m | \mathbf{x}^{\text{obs}}, \mathbf{m})} \right] \right) \\
&\geq \mathbb{E}_{\mathbf{h}_x, \mathbf{h}_m \sim q_\phi} \left(\log \left[\frac{p_\theta(\mathbf{x}^{\text{obs}}, \mathbf{m}, \mathbf{t}, \mathbf{h}_x, \mathbf{h}_m)}{q_\phi(\mathbf{h}_x, \mathbf{h}_m | \mathbf{x}^{\text{obs}}, \mathbf{m})} \right] \right) \\
&= \mathbb{E}_{\mathbf{h}_x, \mathbf{h}_m \sim q_\phi} \left(\log \left[\frac{p_\theta(\mathbf{x}^{\text{obs}} | \mathbf{h}_x, \mathbf{h}_m) p_\theta(\mathbf{m} | \mathbf{h}_m) p_\theta(\mathbf{t} | \mathbf{h}_x, \mathbf{h}_m) p_\theta(\mathbf{h}_x) p_\theta(\mathbf{h}_m)}{q_\phi(\mathbf{h}_x | \mathbf{h}_m, \mathbf{x}^{\text{obs}}) q_\phi(\mathbf{h}_m | \mathbf{m})} \right] \right) \\
&= \underbrace{\mathbb{E}_{\mathbf{h}_x, \mathbf{h}_m \sim q_\phi} \left[\log p_\theta(\mathbf{t} | \mathbf{h}_x, \mathbf{h}_m) \right]}_{\text{Survival Likelihood}} \\
&\quad + \underbrace{\mathbb{E}_{\mathbf{h}_x, \mathbf{h}_m \sim q_\phi} \left[\log p_\theta(\mathbf{x}^{\text{obs}} | \mathbf{h}_x, \mathbf{h}_m) + \log p_\theta(\mathbf{m} | \mathbf{h}_m) \right]}_{\text{Reconstruction}} \\
&\quad - \underbrace{\mathbb{E}_{\mathbf{h}_m \sim q_\phi} \left[\mathrm{KL} \left(q_\phi(\mathbf{h}_x | \mathbf{h}_m, \mathbf{x}^{\text{obs}}) || p_\theta(\mathbf{h}_x) \right) \right] - \mathrm{KL} \left(q_\phi(\mathbf{h}_m | \mathbf{m}) || p_\theta(\mathbf{h}_m) \right)}_{\text{KL Regularisation}}
\end{aligned}
$$

III Simulation

A Simulating Survival Time

The Weibull proportional hazards model is a widely used parametric survival model due to its flexibility in capturing different hazard shapes. This, along with its closed-form expressions, makes it a common choice for simulating survival data. The hazard function is defined as

$$h(t|\mathbf{x}) = \lambda p t^{p-1} \exp(\beta^T \mathbf{x}),$$

where t represents survival time, $\lambda > 0$ is the baseline hazard rate, $p > 0$ is the shape parameter, and β is the corresponding vector of regression coefficients. The survival function follows as

$$S(t|\mathbf{x}) = \exp\left(-\int_t h(u|\mathbf{x})\, du\right) = \exp\left(-\lambda t^p \exp(\beta^T \mathbf{x})\right).$$

Observed event times t_E are generated using inverse transform sampling. Given $F(t|\mathbf{x})$ is a CDF and the survival function is defined as $S(t|\mathbf{x}) = 1 - F(t|\mathbf{x})$, setting

$$S(t_E|\mathbf{x}) = U, \quad U \sim \text{Uniform}(0, 1)$$

yields

$$\exp\left(-\lambda t_E^p \exp(\beta^T \mathbf{x})\right) = U.$$

Solving for t_E results in

$$t_E = \left(\frac{-\log(U)}{\lambda \exp(\beta^T \mathbf{x})}\right)^{1/p}.$$

To simulate right censoring, a maximum follow-up time $t_{\max}$ is introduced with an independent censoring time t_C drawn from an exponential distribution. The observed survival time and event indicator are then

$$T = \min(t_E, t_C, t_{\max}),$$
$$\delta = 1(t_E \leq \min(t_C, t_{\max})).$$

B Simulation Parameters

Step 1: Simulate Latent Variables

$$\begin{pmatrix} H \\ B \end{pmatrix} \sim \mathcal{MVN}\left(\begin{pmatrix} 0 \\ 0 \end{pmatrix}, \begin{pmatrix} 0.09 & 0.10 \\ 0.10 & 0.16 \end{pmatrix}\right) \qquad \rho \approx 0.83$$

Step 2: Simulate Risk Factors

Table 1: Risk Factors

Variable	Distribution
Age	$\mathcal{N}_{truncated}(\mu = 50 - 10 \times H, \sigma = 5, a = 20, b = 80)$
Sex	$\mathcal{B}ernoulli(p = 0.5)$
Smoking Status	$p_{\text{current}} = \max(0, 0.30 - 0.25 \times B)$, $p_{\text{never}} = \max(0, 0.45 - 0.30 \times B)$
	$p_{\text{ex}} = \max(0, 1 - (p_{\text{current}} + p_{\text{never}}))$
	$\mathcal{M}ultinoulli\left(p = \left(\frac{p_{\text{current}}}{total}, \frac{p_{\text{ex}}}{total}, \frac{p_{\text{never}}}{total}\right)\right)$, where $total = p_{\text{current}} + p_{\text{ex}} + p_{\text{never}}$
Prior Condition	$\mathcal{B}ernoulli(p = \frac{1}{1+\exp(H+1)})$
Treatment	$\mathcal{B}ernoulli(p = p)$
	$p = \text{ifelse(Prior Condition} == 1, 0.5 - 0.2 \times H + 0.001 \times (\text{Age} - 20), 0)$
v_1	$\mathcal{N}_{\text{truncated}}(175, 5, 150, 200)$ if Sex = Male
	$\mathcal{N}_{\text{truncated}}(162, 5, 140, 190)$ if Sex = Female
v_2	$\mathcal{N}_{\text{truncated}}(30 - 10 \times H, 5, 15, 60)$
v_3	$\mathcal{N}(130 - 20 \times H, 5)$
v_4	$\mathcal{N}(5.5 - H, 1)$
v_5	$\mathcal{N}(120 + 5 \times H - 0.8 \times Age, 5)$
v_6	$\mathcal{N}(5.5 - 0.5 \times H - 0.1 \times B, 0.5)$
v_7	$\mathcal{N}(25 + 2 \times H, 5)$
v_8	$\mathcal{N}(3 - H, 1)$
v_9	$\mathcal{N}(6 - 0.5 \times H, 1)$
v_{10}	$\mathcal{N}(1.5 + 0.2 \times H, 0.5)$
v_{11}	$\mathcal{N}(400 - 10 \times H, 30)$
v_{12}	$\mathcal{N}(10 - 2 \times H, 3)$
v_{13}	$\mathcal{N}(2 + 0.05 \times H, 0.5)$
v_{14}	$\mathcal{B}ernoulli(p = 0.2 - 0.1 \times H)$
v_{15}	$\mathcal{N}(3 - 0.05 \times H, 0.3)$

Step 3: Simulate Survival Outcome

Table 2: Weibull Proportional Hazards Coefficients

**Covariate**	**Coefficient (β)**	**Covariate**	**Coefficient (β)**
**Weibull Parameters**		**Risk Factors**	
λ	0.0025	v_1	0
p	1.0500	v_2	0.02
**Latent Variables**		v_3	0.03
H (Latent Health)	-0.75	v_4	0.05
B (Latent Behaviour)	-0.25	v_5	-0.05
**Demographics and Lifestyle**		v_6	0.06
Age	0.01	v_7	0
Sex (Female)	-0.1	v_8	0.08
Smoker (Current)	0.3	v_9	0
Smoker (Ex)	0.15	v_{10}	0
Prior Condition	0.5	v_{11}	0.0001
Treatment	-0.2	v_{12}	-0.05
**Interactions & Non-Linear Terms**		v_{13}	0
Age^2	0.0001	v_{14}	0
$H \times \text{Age}$	-0.005	v_{15}	0
$B \times \text{Treatment}$	-0.005		
$v_2 \times v_6$	0.003		
**Event**			$\approx$ **0.25**

Step 4: Induce Missingness

To simulate realistic missingness patterns, we introduce a latent missingness variable Z with the same dimensionality as X, $Z \sim \mathcal{N}(\mu, \Sigma)$.

Table 3: μ values.

**Baseline Missingness** μ_{baseline}	**Shifted Missingness** μ_{shift}
$\mu_1 = -0.5$	Unchanged
$\mu_2 = -0.8 + 0.2 \times H - 0.1 \times B$	Unchanged
$\mu_3 = -0.7 + 1.0 \times H$	Unchanged
$\mu_4 = -0.2 + 2.0 \times H$	$\mu_4 = +0.0 + 1.0 \times H^*$
$\mu_5 = +0.0 + 1.0 \times H - 0.2 \times B$	$\mu_5 = +0.0 + 0.5 \times H^*$
$\mu_6 = -0.1 + 1.5 \times H - 0.2 \times B$	$\mu_6 = -0.1 + 0.2 \times H^*$
$\mu_7 = +1.0 + 1.0 \times H$	$\mu_7 = +0.2 + 1.0 \times H - 0.2 \times B$
$\mu_8 = +0.8 + 1.0 \times H^*$	$\mu_8 = +0.1 + 1.5 \times H^*$
$\mu_9 = +0.7 + 1.0 \times H - 0.5 \times B$	$\mu_9 = +0.0 + 1.5 \times H - 0.8 \times B$
$\mu_{10} = +1.0 + 1.0 \times H$	Unchanged
$\mu_{11} = +1.5 + 1.0 \times H^*$	$\mu_{11} = +0.5 + 2.0 \times H^*$
$\mu_{12} = +1.2 + 1.2 \times H^*$	$\mu_{12} = +0.6 + 1.5 \times H$
$\mu_{13} = +1.8 + 1.0 \times H^* - 0.5 \times B$	Unchanged
$\mu_{14} = +2.0 + 1.0 \times H^* - 0.5 \times B$	Unchanged
$\mu_{15} = +1.0 + 2.0 \times H^*$	Unchanged

H^*: $\text{sign}(H) \times \sqrt{|H|}$

$$
\Sigma_{\text{baseline}} = \begin{pmatrix}
1 & 0.9 & 0.6 & 0 & 0 & 0 & 0 & 0 & 0 & 0 & 0 & 0 & 0 & 0 & 0 \\
0.9 & 1 & 0.7 & 0 & 0 & 0 & 0 & 0 & 0 & 0 & 0 & 0 & 0 & 0 & 0 \\
0.6 & 0.7 & 1 & 0.3 & 0 & 0 & 0 & 0 & 0 & 0 & 0 & 0 & 0 & 0 & 0 \\
0 & 0 & 0.3 & 1 & 0.6 & 0.5 & 0 & 0 & 0 & 0 & 0 & 0 & 0 & 0 & 0 \\
0 & 0 & 0 & 0.6 & 1 & 0.7 & 0 & 0 & 0 & 0 & 0 & 0 & 0 & 0 & 0 \\
0 & 0 & 0 & 0.5 & 0.7 & 1 & 0 & 0 & 0 & 0 & 0 & 0 & 0 & 0 & 0 \\
0 & 0 & 0 & 0 & 0 & 0 & 1 & 0.6 & 0.5 & 0 & 0 & 0 & 0 & 0 & 0 \\
0 & 0 & 0 & 0 & 0 & 0 & 0.6 & 1 & 0.7 & 0 & 0 & 0 & 0 & 0 & 0 \\
0 & 0 & 0 & 0 & 0 & 0 & 0.5 & 0.7 & 1 & 0 & 0 & 0 & 0 & 0 & 0 \\
0 & 0 & 0 & 0 & 0 & 0 & 0 & 0 & 0 & 1 & 0 & 0 & 0 & 0 & 0 \\
0 & 0 & 0 & 0 & 0 & 0 & 0 & 0 & 0 & 0 & 1 & 0.8 & 0 & 0 & 0 \\
0 & 0 & 0 & 0 & 0 & 0 & 0 & 0 & 0 & 0 & 0.8 & 1 & 0 & 0 & 0 \\
0 & 0 & 0 & 0 & 0 & 0 & 0 & 0 & 0 & 0 & 0 & 0 & 1 & 0.7 & 0 \\
0 & 0 & 0 & 0 & 0 & 0 & 0 & 0 & 0 & 0 & 0 & 0 & 0.7 & 1 & 0 \\
0 & 0 & 0 & 0 & 0 & 0 & 0 & 0 & 0 & 0 & 0 & 0 & 0 & 0 & 1
\end{pmatrix}
$$

$$
\Sigma_{\text{shift}} = \begin{pmatrix}
1 & 0.9 & 0.6 & 0 & 0 & 0 & 0 & 0 & 0 & 0 & 0 & 0 & 0 & 0 & 0 \\
0.9 & 1 & 0.7 & 0 & 0 & 0 & 0 & 0 & 0 & 0 & 0 & 0 & 0 & 0 & 0 \\
0.6 & 0.7 & 1 & 0.3 & 0 & 0 & 0 & 0 & 0 & 0 & 0 & 0 & 0 & 0 & 0 \\
0 & 0 & 0.3 & 1 & 0.8 & 0 & 0 & 0 & 0 & 0 & 0 & 0 & 0 & 0 & 0 \\
0 & 0 & 0 & 0.8 & 1 & 0 & 0 & 0 & 0 & 0 & 0 & 0 & 0 & 0 & 0 \\
0 & 0 & 0 & 0 & 0 & 1 & 0.75 & 0 & 0 & 0 & 0 & 0 & 0 & 0 & 0 \\
0 & 0 & 0 & 0 & 0 & 0.75 & 1 & 0 & 0 & 0 & 0 & 0 & 0 & 0 & 0 \\
0 & 0 & 0 & 0 & 0 & 0 & 0 & 1 & 0.85 & 0.75 & 0 & 0 & 0 & 0 & 0 \\
0 & 0 & 0 & 0 & 0 & 0 & 0 & 0.85 & 1 & 0.8 & 0 & 0 & 0 & 0 & 0 \\
0 & 0 & 0 & 0 & 0 & 0 & 0 & 0.75 & 0.8 & 1 & 0 & 0 & 0 & 0 & 0 \\
0 & 0 & 0 & 0 & 0 & 0 & 0 & 0 & 0 & 0 & 1 & 0.75 & 0.6 & 0.5 & 0 \\
0 & 0 & 0 & 0 & 0 & 0 & 0 & 0 & 0 & 0 & 0.75 & 1 & 0.8 & 0.7 & 0 \\
0 & 0 & 0 & 0 & 0 & 0 & 0 & 0 & 0 & 0 & 0.6 & 0.8 & 1 & 0.85 & 0 \\
0 & 0 & 0 & 0 & 0 & 0 & 0 & 0 & 0 & 0 & 0.5 & 0.7 & 0.85 & 1 & 0 \\
0 & 0 & 0 & 0 & 0 & 0 & 0 & 0 & 0 & 0 & 0 & 0 & 0 & 0 & 1
\end{pmatrix}
$$

C Overview of Simulation Scenarios

1. Simulation A: Baseline
2. Simulation B: Unseen missingness patterns
   - Remove all pairs in block 3, i.e.
     - xxxxxxx $\times(1,1,0)\times$ xxxxxx
     - xxxxxxx $\times(1,0,1)\times$ xxxxxx
     - xxxxxxx $\times(0,1,1)\times$ xxxxxx
   - Remove complete pair in block 4, i.e.
     - xxxxxxxxxxx $\times(1,1)\times$ xxx
   - This removes $3 \times 2^{12} + 2^{13} - 3 \times 2^{10} = 17,408$, keeping $15,360$ patterns.
3. Simulation C: Missingness Shift
   - Training Set: Step 4 follows $Z \sim \mathcal{N}(\mu_{\text{shift}}, \Sigma_{\text{shift}})$
   - Test Set: Step 4 follows $Z \sim \mathcal{N}(\mu_{\text{baseline}}, \Sigma_{\text{baseline}})$

IV EHR Datasets

A Dataset Description

Summaries of the variables in each dataset are provided in Tables 4 and 5, as well as in Figure 1.

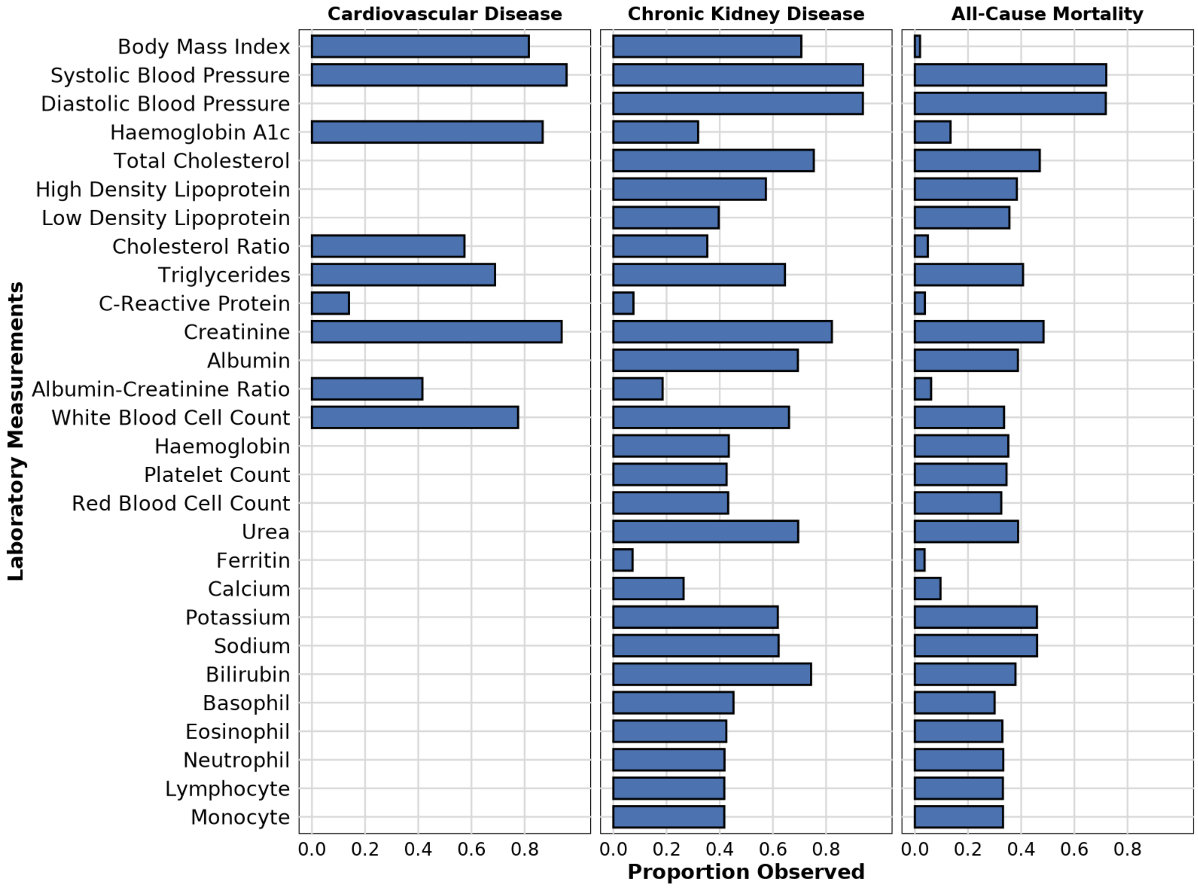

Figure 1: Proportion of observed measurements across different lab measurements.

Table 4: Summary of fully observed variables in the CPRD datasets.

Variables	Cardiovascular Disease		Chronic Kidney Disease		All-Cause Mortality	
	London	North East	London	North East	London	North East
**Total Observations**	36,457	8,084	77,175	21,110	64,586	17,054
**Outcome**						
Event (Mean)	0.0906	0.1176	0.2262	0.2855	0.2183	0.3318
Time in Years (Mean)	5.6456	6.0708	6.0664	7.2112	9.4070	11.4427
**Age at Recruitment (Years)**						
Mean (SD)	68.16 (5.54)	68.43 (5.46)	69.89 (2.87)	69.87 (2.87)	67.40 (1.41)	67.40 (1.41)
**Sex**						
Female	18,488 (50.7%)	4,031 (49.9%)	41,542 (53.8%)	11,276 (53.4%)	32,530 (50.4%)	8,917 (47.7%)
Male	17,969 (49.3%)	4,053 (50.1%)	35,633 (46.2%)	9,834 (46.6%)	32,056 (49.6%)	8,137 (52.3%)
**Ethnicity**						
Asian	7,204 (19.7%)	73 (0.9%)	12,291 (15.9%)	119 (0.6%)	6,033 (9.3%)	56 (0.3%)
Black	5,885 (16.1%)	12 (0.2%)	9,701 (12.6%)	12 (0.1%)	5,291 (8.2%)	3 (0.0%)
Mixed	774 (2.1%)	18 (0.2%)	1,470 (1.9%)	27 (0.1%)	760 (1.2%)	10 (0.1%)
Other	576 (1.6%)	5 (0.1%)	1,110 (1.4%)	14 (0.1%)	416 (0.6%)	9 (0.1%)
White	19,879 (54.6%)	7,408 (91.6%)	43,583 (56.5%)	17,364 (82.2%)	34,786 (53.9%)	13,541 (79.4%)
Missing	2,139 (5.9%)	568 (7.0%)	9,020 (11.7%)	3,574 (16.9%)	17,300 (26.8%)	3,435 (20.1%)
**IMD**						
1	4,343 (10.5%)	738 (9.1%)	8,257 (10.7%)	1,802 (8.6%)	8,616 (13.3%)	1,757 (10.3%)
2	6,604 (15.8%)	1,198 (14.8%)	12,097 (15.7%)	2,878 (13.6%)	10,839 (16.8%)	2,624 (15.4%)
3	7,520 (20.6%)	1,166 (14.4%)	15,740 (20.4%)	2,938 (13.9%)	12,785 (19.8%)	2,557 (15.0%)
4	11,855 (32.5%)	2,033 (25.2%)	22,540 (29.2%)	5,095 (24.1%)	16,966 (26.3%)	3,937 (23.1%)
5	7,315 (19.6%)	2,897 (35.8%)	12,840 (16.6%)	7,290 (34.5%)	9,004 (13.9%)	5,606 (32.9%)
Missing	379 (1.0%)	52 (0.7%)	5,737 (7.4%)	1,107 (5.3%)	6,376 (9.9%)	573 (3.4%)
**Smoking**						
Never	15,068 (41.4%)	2,944 (36.4%)	35,939 (46.6%)	6,924 (32.8%)	1,398 (2.2%)	0 (0.0%)
Current	1,647 (4.5%)	597 (7.4%)	4,434 (5.8%)	1,801 (8.5%)	609 (0.9%)	674 (4.0%)
Ex	7,410 (20.3%)	2,591 (32.1%)	15,479 (20.1%)	5,780 (27.4%)	1,183 (1.8%)	381 (2.2%)
Missing	12,332 (33.8%)	1,952 (24.1%)	21,323 (27.6%)	6,605 (31.3%)	61,396 (95.1%)	15,999 (93.8%)
**Prior Condition**						
Depression	5,506 (15.1%)	1,917 (23.7%)	19,885 (25.8%)	7,126 (33.8%)	6,348 (9.8%)	2,871 (16.8%)
Anxiety	3,828 (10.5%)	1,630 (20.2%)	13,524 (17.5%)	6,240 (29.6%)	4,222 (6.5%)	2,526 (14.8%)
Hypertension	21,399 (58.7%)	4,770 (59.0%)	60,029 (77.8%)	14,836 (70.3%)	26,043 (40.3%)	7,276 (42.7%)
Type 2 Diabetes	36,457 (100.0%)	8,084 (100.0%)	29,509 (38.2%)	4,569 (21.6%)	8,634 (13.4%)	1,697 (10.0%)
Chronic Kidney Disease	2,572 (7.1%)	725 (9.0%)	-	-	231 (0.4%)	31 (0.2%)
Osteoarthritis	-	-	33,383 (43.3%)	12,522 (59.3%)	12,977 (20.1%)	6,199 (36.3%)
All Cancer	-	-	12,559 (16.3%)	3,322 (15.7%)	4,562 (7.1%)	1,341 (7.9%)
Cardiovascular Disease	-	-	26,023 (33.7%)	8,511 (40.3%)	8,679 (13.4%)	3,560 (20.9%)
**Drugs**						
Hypertensive	16,910 (46.4%)	3,923 (48.5%)	44,116 (57.2%)	12,259 (58.1%)	28,292 (43.8%)	8,064 (47.3%)
Glucose Lowering	9,502 (26.1%)	1,329 (16.4%)	23,931 (31.0%)	3,331 (15.8%)	7,595 (11.8%)	1,305 (7.7%)
Statins or Lipid Lowering (incl. Statins)	3,868 (10.6%)	2,032 (25.1%)	9,908 (12.8%)	5,951 (28.2%)	18,635 (28.9%)	5,851 (34.3%)
Corticosteroids	6,725 (18.4%)	2,155 (26.7%)	9,622 (12.5%)	4,073 (19.3%)	5,798 (9.0%)	2,566 (15.0%)
Diuretics	8,214 (22.5%)	2,478 (30.7%)	19,954 (25.9%)	7,256 (34.4%)	21,605 (33.5%)	6,489 (38.0%)
Anticoagulants	531 (1.5%)	199 (2.5%)	830 (1.1%)	217 (1.0%)	0 (0.0%)	0 (0.0%)
Antiplatelet	-	-	16,280 (21.1%)	6,174 (29.2%)	17,152 (26.6%)	5,605 (32.9%)
General and Neuropathic Pain	-	-	39,355 (51.0%)	14,068 (66.6%)	45,015 (69.7%)	13,981 (82.0%)
Bisphosphonates	-	-	4,478 (5.8%)	1,465 (6.9%)	2,584 (4.0%)	754 (4.4%)

Table 5: Summary of partially observed lab measurements in the CPRD datasets.

Lab Measurements	Unit	Min	Max	Cardiovascular Disease		Chronic Kidney Disease		All-Cause Mortality	
				London	North East	London	North East	London	North East
Body Mass Index	kg/m^2	14	75	30.89 (6.16)	32.32 (6.33)	28.67 (5.59)	28.78 (5.50)	28.37 (5.56)	NaN (NA)
Systolic Blood Pressure	mmHg	70	250	137.95 (16.25)	140.01 (16.56)	137.55 (17.00)	138.61 (17.55)	138.60 (16.98)	140.16 (17.19)
Diastolic Blood Pressure	mmHg	40	120	-	-	77.84 (9.57)	76.93 (9.74)	79.73 (9.30)	78.57 (9.40)
Haemoglobin A1c	mmol/mol	15	200	58.45 (19.58)	59.75 (20.06)	53.59 (16.48)	50.00 (14.58)	56.67 (16.76)	52.12 (15.55)
Total Cholesterol	mmol/L	2.6	7.8	-	-	4.72 (1.06)	4.78 (1.06)	5.12 (1.04)	5.05 (1.06)
High Density Lipoprotein	mmol/L	0.5	3.0	-	-	1.38 (0.39)	1.40 (0.40)	1.42 (0.39)	1.42 (0.39)
Low Density Lipoprotein	mmol/L	1.0	6.0	-	-	2.69 (0.92)	2.71 (0.95)	3.06 (0.94)	2.94 (0.98)
Cholesterol Ratio	-	0.2	25.0	4.08 (1.28)	4.12 (1.25)	3.59 (1.12)	3.56 (1.09)	3.66 (1.10)	4.23 (1.24)
Triglycerides	mmol/L	0.1	37.5	1.83 (1.18)	2.32 (1.53)	1.53 (0.89)	1.81 (1.05)	1.54 (0.89)	1.83 (1.07)
C-Reactive Protein	mg/L	0	200	10.53 (20.14)	13.47 (23.19)	9.49 (19.39)	10.91 (21.99)	10.30 (19.25)	12.22 (21.33)
Creatinine	μmol/L	1	1000	84.11 (24.59)	84.76 (23.96)	83.53 (33.54)	90.96 (29.24)	92.66 (33.10)	91.83 (25.81)
Albumin	g/L	20	60	-	-	41.78 (3.73)	42.90 (3.35)	41.58 (3.36)	43.18 (3.15)
Albumin-Creatinine Ratio	mg/mmol	0	200	4.39 (12.80)	2.78 (8.24)	6.34 (17.53)	3.66 (10.74)	5.46 (15.27)	3.18 (8.42)
White Blood Cell Count	10^9/L	1	50	7.53 (2.37)	7.87 (2.38)	7.29 (2.35)	7.43 (2.33)	7.15 (2.23)	7.30 (2.39)
Haemoglobin	g/L	90	180	-	-	135.30 (14.62)	137.77 (14.14)	138.39 (14.11)	139.54 (13.86)
Platelet Count	10^9/L	100	500	-	-	254.85 (67.89)	260.66 (68.27)	254.02 (65.54)	264.03 (67.88)
Red Blood Cell Count	10^12/L	3.5	6.5	-	-	4.58 (0.47)	4.55 (0.44)	4.60 (0.46)	4.57 (0.42)
Urea	mmol/L	1.0	15.0	-	-	5.99 (1.92)	6.09 (1.89)	5.80 (1.76)	5.96 (1.76)
Ferritin	μg/L	10	800	-	-	118.44 (120.83)	114.91 (121.65)	114.25 (114.83)	117.55 (128.15)
Calcium	mmol/L	1.5	3.0	-	-	2.36 (0.12)	2.37 (0.11)	2.35 (0.12)	2.38 (0.11)
Potassium	mmol/L	2.5	6.5	-	-	4.46 (0.48)	4.30 (0.46)	4.43 (0.47)	4.30 (0.46)
Sodium	mmol/L	120	160	-	-	140.04 (2.96)	139.52 (3.01)	139.87 (2.74)	139.38 (2.90)
Bilirubin	μmol/L	0	50	-	-	10.54 (5.24)	10.16 (5.09)	11.37 (5.27)	10.66 (5.09)
Basophil	10^9/L	0.0	0.2	-	-	0.04 (0.04)	0.04 (0.04)	0.03 (0.04)	0.04 (0.03)
Eosinophil	10^9/L	0	1	-	-	0.22 (0.16)	0.21 (0.14)	0.21 (0.15)	0.20 (0.14)
Neutrophil	10^9/L	0.5	10.0	-	-	4.19 (1.54)	4.33 (1.50)	4.09 (1.53)	4.19 (1.48)
Lymphocyte	10^9/L	0.5	5.0	-	-	2.18 (0.74)	2.13 (0.73)	2.17 (0.70)	2.17 (0.71)
Monocyte	10^9/L	0.1	1.5	-	-	0.54 (0.20)	0.59 (0.21)	0.50 (0.19)	0.55 (0.21)

B Overview of Experimental Scenarios

1. Baseline (Train and Test on London)
2. Unseen missingness patterns
   - CVD Dataset – Remove counts of 2 '1s' in HbA1c, Creatinine, White Blood Cell Count
     - $... \times (1, 1, 0) \times ...$
     - $... \times (1, 0, 1) \times ...$
     - $... \times (0, 1, 1) \times ...$
   - CKD Dataset – Remove counts of 3 or 4 '1s' in Total Cholesterol, Cholesterol Ratio, High Density Lipoprotein, Low Density Lipoprotein, Triglycerides
     - $... \times (0, 0, 1, 1, 1) \times ...$
     - $... \times (0, 1, 0, 1, 1) \times ...$
     - $... \times (0, 1, 1, 0, 1) \times ...$
     - $... \times (0, 1, 1, 1, 0) \times ...$
     - $... \times (0, 1, 1, 1, 1) \times ...$
     - $... \times (1, 0, 0, 1, 1) \times ...$
     - $... \times (1, 0, 1, 0, 1) \times ...$
     - $... \times (1, 0, 1, 1, 0) \times ...$
     - $... \times (1, 0, 1, 1, 1) \times ...$
     - $... \times (1, 1, 0, 0, 1) \times ...$
     - $... \times (1, 1, 0, 1, 0) \times ...$
     - $... \times (1, 1, 0, 1, 1) \times ...$
     - $... \times (1, 1, 1, 0, 0) \times ...$
     - $... \times (1, 1, 1, 0, 1) \times ...$
     - $... \times (1, 1, 1, 1, 0) \times ...$
   - ACM Dataset – Similar to CKD dataset. Remove counts of 3 or 4 '1s' in HbA1c, Creatinine, Albumin-to-Creatinine Ratio, C-Reactive Protein, Low Density Lipoprotein
3. Distribution Shift (Train on London, Test on North East)
   - The standardised mean differences between the training and test sets are presented in Table 6.

Table 6: Standardised mean differences (mean (SD) over 5 folds). For each experiment, we compare the training data with two test sets: a *baseline* test set obtained from the same dataset via 5-fold cross-validation (London patients), and a *shift* test set consisting of patients from a different health authorisation (North East).

**Variables**	**CKD**		**CVD**		**ACM**	
	**Baseline**	**Shift**	**Baseline**	**Shift**	**Baseline**	**Shift**
**Survival Outcome**						
Event	0.007 (0.006)	0.135 (0.002)	0.011 (0.008)	0.089 (0.003)	0.010 (0.012)	0.256 (0.004)
Time	0.005 (0.005)	0.211 (0.001)	0.008 (0.007)	0.098 (0.003)	0.006 (0.003)	0.401 (0.002)
**Lab Measurements**						
Body Mass Index	0.006 (0.001)	0.021 (0.002)	0.011 (0.009)	0.229 (0.005)	0.080 (0.064)	-
Systolic Blood Pressure	0.005 (0.002)	0.064 (0.001)	0.014 (0.012)	0.126 (0.003)	0.006 (0.003)	0.090 (0.001)
Diastolic Blood Pressure	0.006 (0.005)	0.095 (0.003)	-	-	0.013 (0.008)	0.126 (0.004)
Haemoglobin A1c	0.010 (0.007)	0.231 (0.003)	0.009 (0.007)	0.067 (0.005)	0.023 (0.017)	0.281 (0.009)
Total Cholesterol	0.009 (0.008)	0.057 (0.004)	-	-	0.011 (0.005)	0.060 (0.003)
High Density Lipoprotein	0.006 (0.005)	0.056 (0.001)	-	-	0.005 (0.002)	0.019 (0.003)
Low Density Lipoprotein	0.014 (0.007)	0.017 (0.004)	-	-	0.014 (0.009)	0.116 (0.003)
Cholesterol Ratio	0.011 (0.004)	0.027 (0.003)	0.010 (0.009)	0.036 (0.005)	0.044 (0.033)	0.499 (0.013)
Triglycerides	0.005 (0.002)	0.284 (0.003)	0.013 (0.008)	0.353 (0.008)	0.010 (0.010)	0.299 (0.006)
C-Reactive Protein	0.010 (0.008)	0.068 (0.007)	0.021 (0.014)	0.141 (0.007)	0.030 (0.012)	0.100 (0.014)
Creatinine	0.005 (0.004)	0.043 (0.003)	0.015 (0.012)	0.026 (0.006)	0.010 (0.006)	0.030 (0.004)
Albumin	0.021 (0.008)	0.313 (0.006)	-	-	0.020 (0.014)	0.491 (0.004)
Albumin-Creatinine Ratio	0.014 (0.018)	0.186 (0.004)	0.013 (0.006)	0.147 (0.006)	0.029 (0.015)	0.183 (0.011)
White Blood Cell Count	0.011 (0.008)	0.058 (0.004)	0.014 (0.009)	0.145 (0.006)	0.018 (0.014)	0.065 (0.005)
Haemoglobin	0.013 (0.005)	0.171 (0.004)	-	-	0.019 (0.008)	0.083 (0.004)
Platelet Count	0.011 (0.009)	0.085 (0.004)	-	-	0.005 (0.004)	0.149 (0.003)
Red Blood Cell Count	0.011 (0.006)	0.070 (0.004)	-	-	0.011 (0.006)	0.081 (0.002)
Urea	0.007 (0.004)	0.050 (0.004)	-	-	0.020 (0.013)	0.093 (0.007)
Ferritin	0.014 (0.012)	0.026 (0.008)	-	-	0.023 (0.029)	0.033 (0.013)
Calcium	0.019 (0.016)	0.057 (0.006)	-	-	0.045 (0.032)	0.276 (0.012)
Potassium	0.004 (0.005)	0.339 (0.004)	-	-	0.011 (0.007)	0.277 (0.005)
Sodium	0.018 (0.013)	0.176 (0.006)	-	-	0.014 (0.011)	0.177 (0.004)
Bilirubin	0.010 (0.008)	0.074 (0.004)	-	-	0.019 (0.010)	0.134 (0.007)
Basophil	0.009 (0.005)	0.090 (0.002)	-	-	0.010 (0.006)	0.085 (0.008)
Eosinophil	0.009 (0.004)	0.066 (0.003)	-	-	0.015 (0.010)	0.056 (0.007)
Neutrophil	0.016 (0.007)	0.094 (0.005)	-	-	0.019 (0.014)	0.068 (0.005)
Lymphocyte	0.018 (0.007)	0.071 (0.004)	-	-	0.010 (0.006)	0.006 (0.005)
Monocyte	0.012 (0.008)	0.255 (0.004)	-	-	0.014 (0.007)	0.255 (0.008)

V Experiments

A Libraries and Packages

Imputation is performed using the `scikit-learn` Python library, specifically employing the `LinearRegressionImputer` and `IterativeImputer` with default settings.[10] For multiple imputation, we use the `mice` package in R.[11] Model training is done using the PyTorch Lightning.[12] Survival metrics are computed with the `pycox` Python library.[8]

B Experimental Setup

We provide the full configuration used across all experiments to support reproducibility in Table 7. For metrics requiring time integration (IBS, INBLL, and ISE), we evaluate on a discrete time grid from $t = 0$ to $t_{\max}$ with step size 0.1.

We use a fixed split of 64% for training, 16% for validation, and 20% for testing. All preprocessing steps are fitted on the training set only and then applied to the validation and test sets. For all non–MICE imputers, the imputation model is fitted on the training set and subsequently applied to the validation and test sets. The exception is MICE, which is jointly fitted on the training and validation sets and at test time, the learned regression models are used to impute missing values. This reflects realistic deployment, since MICE requires refitting if applied to new data containing missingness.

Survival head (DeSurv) hyperparameters follow the defaults of the original paper.[9] MissCVAE architecture dimensions (encoder, decoder, latent) are set proportionally to input dimensionality and number of missingness patterns. No systematic hyperparameter search was performed for any method to ensure a fair comparison. We instead conduct an ablation study (Section V.C and V.F) to assess the sensitivity of MissCVAE to its key architectural choices, which we consider more informative than exhaustive tuning given the moderate model complexity and the number of baseline comparisons.

Table 7: Experimental Configuration Across Datasets

Experiments	Simulations	CKD	CVD	ACM
**Reproducibility**				
Random seeds	Seed = 42 for sklearn KFold, NumPy, PyTorch, CUDA			
**Training Details**				
Optimiser	Adam (learning rate = 1e−4, betas = (0.9, 0.999), eps = 1e−8)			
Batch size	128			
Dropout rate	0.2			
Training epochs	300	300	200	300
Early stopping patience	30	30	20	30
**Survival Head (DeSurv) Architecture**				
Hidden layers	[32, 32]			
**MissCVAE Architecture**				
Encoder hidden layers	[32, 16]	[32, 32]	[32, 16]	[32, 32]
Decoder hidden layers	[16, 32]	[32, 32]	[16, 32]	[32, 32]
Latent dimension ($\mathbf{h}_m$)	8	32	4	32
Latent dimension ($\mathbf{h}_x$)	8	32	8	32
**Evaluation Settings**				
Max time	20.0	17.5	17.5	15.0

C Ablation Study

An ablation study isolates the effects of individual components and hyperparameter choices, providing insights into the robustness and generalisation of **MissCVAE**. The baseline ELBO weights in the ablation are $\alpha = 2$, $\boldsymbol{\beta} = (\beta_M, \beta_X)$ and $\beta_M = \beta_X = 0.1$, $\boldsymbol{\gamma} = (\gamma_M, \gamma_X)$ and $\gamma_M = \gamma_X = 0.1$. The ablation configurations are summarised in Table 8.

**Ablation Configuration**	**Description**
$\mathbf{h}_x$ only	Removes $\mathbf{h}_m$ as input to the DeSurv module to assess the importance of the missingness representation in prediction.
$\alpha = 1, 5, 10$	Evaluate the impact of a weaker or stronger emphasis on survival loss on prediction performance.
$\beta = 0.05, 0.2$	Evaluate the impact of a weaker or stronger emphasis on reconstruction loss on prediction performance.
$\gamma = 0.05, 0.2$	Evaluate whether a weaker or stronger emphasis on KL divergences improves performance or results in posterior collapse.
[1]$\dim(\mathbf{h}_m), \dim(\mathbf{h}_x) = [4,4], [16,16]$<br>[2]$\dim(\mathbf{h}_m), \dim(\mathbf{h}_x) = [16,16], [64,64]$	Evaluate the impact of varying the latent space dimension, assessing the trade-off between representation capacity and overparameterisation.

[1] Simulation and CVD experiments.
[2] CKD and ACM experiments.

Table 8: Ablation configurations for evaluating the components of **MissCVAE**.

D Additional Simulation Results

Table 9 reports the results for Simulation B evaluated on the full dataset.

Simulation B: Unseen Missingness Patterns (Full)

**Experiment**	$\mathbf{C^{td}} \uparrow$	**IBS** $\downarrow$	**INBLL** $\downarrow$	**-Loglikelihood** $\downarrow$	**ISE** $\downarrow$
CW	0.699 (0.004)	0.161 (0.001)	0.487 (0.003)	0.257 (0.004)	0.520 (0.006)
CW + M	0.721 (0.004)	0.154 (0.002)	0.470 (0.004)	0.240 (0.005)	0.380 (0.006)
Mean	0.746 (0.004)	**0.144 (0.003)**	0.445 (0.006)	0.214 (0.006)	0.187 (0.003)
ICE	0.739 (0.008)	0.149 (0.004)	0.483 (0.046)	0.271 (0.081)	0.271 (0.082)
RI	0.747 (0.004)	**0.144 (0.002)**	0.444 (0.006)	0.213 (0.006)	0.176 (0.003)
MICE O	0.745 (0.003)	0.145 (0.002)	0.445 (0.005)	0.215 (0.005)	0.187 (0.004)
MICE	0.743 (0.003)	0.146 (0.002)	0.448 (0.004)	0.218 (0.005)	0.209 (0.002)
-1 Enc.	0.740 (0.004)	0.147 (0.002)	0.451 (0.005)	0.221 (0.006)	0.235 (0.008)
MIM	**0.748 (0.004)**	**0.144 (0.002)**	**0.443 (0.006)**	**0.212 (0.007)**	**0.168 (0.007)**
MissCVAE	**0.749 (0.003)**	**0.143 (0.002)**	**0.442 (0.004)**	**0.212 (0.005)**	**0.164 (0.006)**

Table 9: Comparison of model performance in the simulation studies, reported as mean (standard deviation) over 5 simulations.

E Additional Experimental Results

Cardiovascular Disease

Experiment	Baseline				Unseen Missingness Patterns (Full)			
	$\mathbf{C^{td}}$ ↑	**IBS** ↓	**INBLL** ↓	**-Likelihood** ↓	$\mathbf{C^{td}}$ ↑	**IBS** ↓	**INBLL** ↓	**-Likelihood** ↓
CW	0.580 (0.010)	**0.113 (0.002)**	**0.369 (0.006)**	0.202 (0.004)	0.578 (0.012)	**0.113 (0.002)**	0.370 (0.005)	0.203 (0.004)
CW + M	0.586 (0.015)	**0.113 (0.002)**	**0.369 (0.006)**	0.202 (0.004)	0.581 (0.016)	**0.113 (0.002)**	**0.369 (0.005)**	**0.202 (0.003)**
Mean	0.590 (0.012)	0.114 (0.002)	0.371 (0.005)	**0.201 (0.004)**	0.588 (0.014)	0.114 (0.002)	0.372 (0.005)	**0.202 (0.003)**
ICE	**0.592 (0.011)**	0.114 (0.002)	0.371 (0.005)	**0.201 (0.004)**	**0.590 (0.012)**	0.114 (0.002)	0.372 (0.005)	**0.202 (0.003)**
RI	0.591 (0.013)	0.114 (0.002)	0.371 (0.005)	**0.201 (0.004)**	0.589 (0.015)	0.114 (0.002)	0.372 (0.005)	**0.202 (0.003)**
MICE O	0.590 (0.012)	0.114 (0.002)	0.370 (0.005)	0.202 (0.003)	0.589 (0.013)	0.114 (0.002)	0.371 (0.004)	**0.202 (0.003)**
MICE	0.590 (0.012)	**0.113 (0.002)**	0.370 (0.005)	0.202 (0.003)	0.589 (0.014)	0.114 (0.002)	0.371 (0.004)	**0.202 (0.003)**
-1 Enc.	0.581 (0.014)	**0.113 (0.002)**	**0.369 (0.005)**	0.202 (0.004)	0.578 (0.012)	0.114 (0.002)	0.371 (0.005)	0.203 (0.004)
MIM	0.591 (0.010)	0.114 (0.002)	0.371 (0.005)	**0.201 (0.003)**	0.589 (0.009)	0.114 (0.002)	0.372 (0.004)	**0.202 (0.003)**
MissCVAE	0.591 (0.008)	**0.113 (0.002)**	**0.369 (0.005)**	0.202 (0.004)	**0.592 (0.010)**	**0.113 (0.002)**	0.370 (0.005)	0.204 (0.004)
MissCVAE ($\mathbf{h}_x$)	0.592 (0.007)	**0.113 (0.002)**	**0.369 (0.006)**	0.202 (0.004)	**0.592 (0.010)**	**0.113 (0.002)**	**0.369 (0.006)**	0.203 (0.004)

Chronic Kidney Disease

Experiment	Baseline				Unseen Missingness Patterns (Full)			
	$\mathbf{C^{td}}$ ↑	**IBS** ↓	**INBLL** ↓	**-Likelihood** ↓	$\mathbf{C^{td}}$ ↑	**IBS** ↓	**INBLL** ↓	**-Likelihood** ↓
CW	0.620 (0.008)	0.168 (0.002)	0.505 (0.004)	0.269 (0.003)	0.624 (0.003)	0.169 (0.001)	0.507 (0.003)	0.293 (0.003)
CW + M	0.634 (0.005)	0.169 (0.002)	0.508 (0.004)	0.286 (0.009)	0.636 (0.005)	0.170 (0.001)	0.508 (0.003)	0.294 (0.001)
Mean	0.783 (0.006)	0.137 (0.002)	0.425 (0.004)	0.203 (0.004)	0.780 (0.004)	0.139 (0.001)	0.429 (0.003)	0.207 (0.003)
ICE	0.787 (0.005)	**0.136 (0.002)**	0.423 (0.004)	**0.198 (0.003)**	0.784 (0.004)	0.138 (0.001)	0.427 (0.003)	0.203 (0.002)
RI	0.787 (0.005)	**0.136 (0.002)**	0.423 (0.004)	0.199 (0.004)	**0.786 (0.004)**	**0.137 (0.002)**	**0.426 (0.004)**	**0.202 (0.003)**
MICE O	0.785 (0.004)	0.137 (0.002)	0.424 (0.004)	0.203 (0.004)	0.783 (0.004)	0.138 (0.001)	0.427 (0.003)	0.206 (0.003)
MICE	0.783 (0.005)	0.138 (0.002)	0.426 (0.004)	0.203 (0.003)	0.781 (0.004)	0.139 (0.001)	0.430 (0.003)	0.208 (0.003)
-1 Enc.	0.781 (0.005)	0.137 (0.002)	0.426 (0.005)	0.200 (0.007)	0.772 (0.004)	0.141 (0.001)	0.436 (0.003)	0.214 (0.003)
MIM	**0.789 (0.004)**	**0.136 (0.002)**	**0.422 (0.005)**	**0.198 (0.004)**	**0.786 (0.004)**	0.138 (0.002)	0.427 (0.006)	0.203 (0.004)
MissCVAE	0.787 (0.005)	**0.136 (0.002)**	0.423 (0.005)	**0.188 (0.017)**	**0.786 (0.004)**	0.138 (0.001)	**0.426 (0.003)**	0.203 (0.003)
MissCVAE ($\mathbf{h}_x$)	0.787 (0.005)	**0.136 (0.002)**	**0.422 (0.005)**	**0.173 (0.009)**	**0.786 (0.004)**	0.138 (0.001)	**0.426 (0.003)**	**0.200 (0.004)**

All-Cause Mortality

Experiment	Baseline				Unseen Missingness Patterns (Full)			
	$\mathbf{C^{td}}$ ↑	**IBS** ↓	**INBLL** ↓	**-Likelihood** ↓	$\mathbf{C^{td}}$ ↑	**IBS** ↓	**INBLL** ↓	**-Likelihood** ↓
CW	0.695 (0.003)	0.096 (0.002)	0.315 (0.005)	0.358 (0.007)	0.693 (0.003)	0.097 (0.002)	0.316 (0.005)	0.365 (0.007)
CW + M	0.695 (0.003)	0.097 (0.002)	0.316 (0.006)	0.363 (0.008)	0.694 (0.004)	0.097 (0.002)	0.317 (0.007)	0.374 (0.008)
Mean	0.705 (0.003)	0.095 (0.002)	0.311 (0.006)	0.361 (0.007)	0.697 (0.004)	**0.096 (0.002)**	0.316 (0.006)	0.374 (0.008)
ICE	0.706 (0.003)	0.095 (0.002)	0.311 (0.006)	0.357 (0.007)	**0.699 (0.004)**	**0.096 (0.002)**	0.315 (0.006)	**0.371 (0.007)**
RI	0.706 (0.004)	0.095 (0.002)	0.311 (0.006)	**0.358 (0.006)**	0.697 (0.004)	**0.096 (0.002)**	0.316 (0.007)	0.373 (0.008)
MICE O	0.698 (0.003)	0.096 (0.002)	0.315 (0.006)	0.369 (0.009)	0.689 (0.004)	0.097 (0.002)	0.319 (0.005)	0.382 (0.006)
MICE	0.701 (0.004)	0.095 (0.002)	0.312 (0.005)	0.364 (0.007)	0.695 (0.004)	**0.096 (0.002)**	0.315 (0.005)	0.375 (0.006)
-1 Enc.	0.699 (0.003)	0.095 (0.002)	0.312 (0.006)	0.360 (0.007)	0.697 (0.003)	**0.096 (0.002)**	**0.314 (0.005)**	**0.371 (0.007)**
MIM	**0.708 (0.005)**	**0.094 (0.002)**	**0.309 (0.007)**	0.364 (0.008)	**0.699 (0.003)**	**0.096 (0.002)**	0.316 (0.005)	0.377 (0.006)
MissCVAE	0.706 (0.004)	**0.094 (0.002)**	**0.309 (0.006)**	**0.358 (0.007)**	**0.699 (0.002)**	**0.095 (0.002)**	**0.313 (0.006)**	**0.371 (0.007)**
MissCVAE ($\mathbf{h}_x$)	0.707 (0.005)	**0.094 (0.002)**	**0.309 (0.006)**	**0.356 (0.007)**	**0.701 (0.003)**	**0.095 (0.002)**	**0.313 (0.006)**	**0.369 (0.007)**

Table 10: Comparison of model performance in the experimental data, reported as mean (standard deviation) over 5 folds.

F Ablation Results

Simulation A - Baseline

Experiment	$\mathbf{C^{td}}$ ↑	IBS ↓	INBLL ↓	-Likelihood ↓	ISE ↓
MissCVAE	0.749 (0.003)	0.144 (0.002)	0.443 (0.004)	0.212 (0.005)	0.165 (0.006)
$\hookrightarrow h_x$	0.747 (0.003)	0.144 (0.002)	0.443 (0.004)	0.212 (0.005)	0.172 (0.006)
$\hookrightarrow \alpha = 1$	0.748 (0.004)	0.144 (0.002)	0.444 (0.004)	0.214 (0.005)	0.172 (0.008)
$\hookrightarrow \alpha = 5$	0.750 (0.003)	0.143 (0.002)	0.442 (0.004)	0.212 (0.005)	0.160 (0.006)
$\hookrightarrow \alpha = 10$	0.750 (0.003)	0.143 (0.002)	0.442 (0.005)	0.211 (0.005)	0.158 (0.005)
$\hookrightarrow \beta = 0.05$	0.749 (0.003)	0.143 (0.002)	0.442 (0.004)	0.212 (0.005)	0.164 (0.006)
$\hookrightarrow \beta = 0.2$	0.749 (0.004)	0.144 (0.002)	0.443 (0.005)	0.213 (0.007)	0.166 (0.006)
$\hookrightarrow \gamma = 0.05$	0.749 (0.003)	0.143 (0.002)	0.442 (0.004)	0.212 (0.005)	0.164 (0.004)
$\hookrightarrow \gamma = 0.2$	0.748 (0.004)	0.144 (0.002)	0.444 (0.005)	0.213 (0.006)	0.173 (0.008)
$\hookrightarrow$ dim S	0.747 (0.004)	0.144 (0.002)	0.444 (0.004)	0.213 (0.005)	0.175 (0.004)
$\hookrightarrow$ dim L	0.749 (0.003)	0.144 (0.002)	0.443 (0.004)	0.213 (0.005)	0.164 (0.004)

Simulation B - Unseen Missingness Patterns (Full)

Experiment	$\mathbf{C^{td}}$ ↑	IBS ↓	INBLL ↓	-Likelihood ↓	ISE ↓
MissCVAE	0.749 (0.003)	0.144 (0.002)	0.443 (0.005)	0.213 (0.006)	0.168 (0.004)
$\hookrightarrow h_x$	0.747 (0.004)	0.144 (0.002)	0.445 (0.004)	0.214 (0.004)	0.181 (0.006)
$\hookrightarrow \alpha = 1$	0.748 (0.004)	0.144 (0.002)	0.444 (0.005)	0.214 (0.006)	0.176 (0.005)
$\hookrightarrow \alpha = 5$	0.749 (0.003)	0.144 (0.002)	0.443 (0.004)	0.213 (0.005)	0.167 (0.005)
$\hookrightarrow \alpha = 10$	0.749 (0.003)	0.143 (0.002)	0.442 (0.004)	0.212 (0.005)	0.164 (0.006)
$\hookrightarrow \beta = 0.05$	0.749 (0.003)	0.144 (0.002)	0.443 (0.005)	0.213 (0.006)	0.169 (0.006)
$\hookrightarrow \beta = 0.2$	0.748 (0.004)	0.144 (0.002)	0.443 (0.005)	0.213 (0.006)	0.169 (0.002)
$\hookrightarrow \gamma = 0.05$	0.749 (0.003)	0.144 (0.002)	0.443 (0.004)	0.213 (0.005)	0.167 (0.003)
$\hookrightarrow \gamma = 0.2$	0.748 (0.004)	0.144 (0.002)	0.444 (0.005)	0.214 (0.006)	0.177 (0.003)
$\hookrightarrow$ dim S	0.747 (0.004)	0.144 (0.002)	0.445 (0.004)	0.214 (0.005)	0.180 (0.004)
$\hookrightarrow$ dim L	0.749 (0.004)	0.144 (0.002)	0.443 (0.004)	0.213 (0.005)	0.167 (0.004)

Simulation B - Unseen Missingness Patterns (Subset)

Experiment	$\mathbf{C^{td}}$ ↑	IBS ↓	INBLL ↓	-Likelihood ↓	ISE ↓
MissCVAE	0.728 (0.008)	0.158 (0.001)	0.478 (0.003)	0.168 (0.010)	0.201 (0.011)
$\hookrightarrow h_x$	0.727 (0.007)	0.159 (0.002)	0.481 (0.005)	0.170 (0.009)	0.223 (0.020)
$\hookrightarrow \alpha = 1$	0.728 (0.007)	0.159 (0.001)	0.480 (0.004)	0.171 (0.009)	0.212 (0.014)
$\hookrightarrow \alpha = 5$	0.728 (0.008)	0.158 (0.001)	0.477 (0.004)	0.168 (0.010)	0.197 (0.008)
$\hookrightarrow \alpha = 10$	0.728 (0.008)	0.158 (0.001)	0.477 (0.003)	0.168 (0.010)	0.196 (0.010)
$\hookrightarrow \beta = 0.05$	0.728 (0.007)	0.158 (0.001)	0.478 (0.003)	0.168 (0.010)	0.201 (0.011)
$\hookrightarrow \beta = 0.2$	0.727 (0.008)	0.158 (0.002)	0.478 (0.004)	0.169 (0.011)	0.203 (0.010)
$\hookrightarrow \gamma = 0.05$	0.728 (0.008)	0.158 (0.002)	0.478 (0.004)	0.169 (0.011)	0.199 (0.008)
$\hookrightarrow \gamma = 0.2$	0.728 (0.007)	0.159 (0.001)	0.480 (0.003)	0.170 (0.010)	0.214 (0.011)
$\hookrightarrow$ dim S	0.727 (0.008)	0.159 (0.002)	0.482 (0.005)	0.171 (0.010)	0.224 (0.013)
$\hookrightarrow$ dim L	0.728 (0.007)	0.158 (0.001)	0.478 (0.003)	0.169 (0.010)	0.201 (0.011)

Simulation C - Missingness Shift

Experiment	$\mathbf{C^{td}}$ ↑	IBS ↓	INBLL ↓	-Likelihood ↓	ISE ↓
MissCVAE	0.749 (0.004)	0.144 (0.002)	0.443 (0.004)	0.213 (0.005)	0.163 (0.006)
$\hookrightarrow h_x$	0.748 (0.003)	0.144 (0.002)	0.444 (0.004)	0.212 (0.005)	0.171 (0.006)
$\hookrightarrow \alpha = 1$	0.748 (0.004)	0.144 (0.002)	0.444 (0.004)	0.214 (0.005)	0.171 (0.004)
$\hookrightarrow \alpha = 5$	0.749 (0.004)	0.144 (0.002)	0.443 (0.004)	0.212 (0.006)	0.162 (0.005)
$\hookrightarrow \alpha = 10$	0.749 (0.004)	0.144 (0.002)	0.443 (0.004)	0.213 (0.006)	0.163 (0.006)
$\hookrightarrow \beta = 0.05$	0.749 (0.004)	0.144 (0.002)	0.443 (0.004)	0.213 (0.006)	0.165 (0.007)
$\hookrightarrow \beta = 0.2$	0.748 (0.004)	0.144 (0.002)	0.443 (0.005)	0.213 (0.006)	0.165 (0.005)
$\hookrightarrow \gamma = 0.05$	0.749 (0.004)	0.144 (0.002)	0.443 (0.005)	0.212 (0.006)	0.163 (0.005)
$\hookrightarrow \gamma = 0.2$	0.748 (0.004)	0.144 (0.002)	0.444 (0.004)	0.214 (0.005)	0.172 (0.005)
$\hookrightarrow$ dim S	0.748 (0.004)	0.144 (0.002)	0.444 (0.003)	0.214 (0.005)	0.173 (0.006)
$\hookrightarrow$ dim L	0.749 (0.004)	0.144 (0.002)	0.444 (0.004)	0.213 (0.006)	0.169 (0.004)

Table 11: MissCVAE simulation experiments ablation study results.

Cardiovascular Disease

Experiment	Baseline				Distribution Shift			
	C^{td} $\uparrow$	IBS $\downarrow$	INBLL $\downarrow$	-Likelihood $\downarrow$	C^{td} $\uparrow$	IBS $\downarrow$	INBLL $\downarrow$	-Likelihood $\downarrow$
MissCVAE	0.588 (0.006)	0.114 (0.002)	0.370 (0.005)	0.202 (0.004)	0.566 (0.002)	0.128 (0.000)	0.406 (0.001)	0.242 (0.000)
$\hookrightarrow h_x$	0.589 (0.007)	0.114 (0.002)	0.370 (0.006)	0.202 (0.004)	0.566 (0.003)	0.128 (0.000)	0.406 (0.001)	0.242 (0.000)
$\hookrightarrow \alpha = 1$	0.584 (0.005)	0.114 (0.002)	0.371 (0.006)	0.203 (0.004)	0.558 (0.003)	0.128 (0.000)	0.407 (0.001)	0.242 (0.000)
$\hookrightarrow \alpha = 5$	0.592 (0.007)	0.113 (0.002)	0.369 (0.005)	0.202 (0.004)	0.573 (0.003)	0.127 (0.000)	0.403 (0.001)	0.241 (0.000)
$\hookrightarrow \alpha = 10$	0.591 (0.008)	0.113 (0.002)	0.369 (0.005)	0.202 (0.004)	0.574 (0.004)	0.126 (0.000)	0.402 (0.001)	0.241 (0.000)
$\hookrightarrow \beta = 0.05$	0.590 (0.007)	0.113 (0.002)	0.369 (0.006)	0.202 (0.004)	0.574 (0.003)	0.127 (0.000)	0.404 (0.001)	0.241 (0.000)
$\hookrightarrow \beta = 0.2$	0.582 (0.006)	0.114 (0.002)	0.372 (0.005)	0.203 (0.004)	0.556 (0.002)	0.128 (0.000)	0.408 (0.001)	0.243 (0.000)
$\hookrightarrow \gamma = 0.05$	0.588 (0.005)	0.114 (0.002)	0.370 (0.005)	0.202 (0.004)	0.565 (0.003)	0.128 (0.000)	0.406 (0.001)	0.242 (0.000)
$\hookrightarrow \gamma = 0.2$	0.588 (0.006)	0.114 (0.002)	0.371 (0.006)	0.203 (0.004)	0.568 (0.003)	0.128 (0.000)	0.406 (0.001)	0.242 (0.000)
$\hookrightarrow$ dim S	0.589 (0.005)	0.114 (0.003)	0.371 (0.006)	0.203 (0.004)	0.564 (0.002)	0.128 (0.000)	0.407 (0.001)	0.242 (0.000)
$\hookrightarrow$ dim L	0.587 (0.005)	0.114 (0.002)	0.371 (0.005)	0.203 (0.004)	0.563 (0.003)	0.127 (0.000)	0.405 (0.001)	0.242 (0.000)

Cardiovascular Disease – Unseen Missingness Patterns

Experiment	Full Test Set				Subset of Unseen Patterns Only			
	C^{td} $\uparrow$	IBS $\downarrow$	INBLL $\downarrow$	-Likelihood $\downarrow$	C^{td} $\uparrow$	IBS $\downarrow$	INBLL $\downarrow$	-Likelihood $\downarrow$
MissCVAE	0.590 (0.006)	0.113 (0.002)	0.370 (0.005)	0.203 (0.004)	0.577 (0.018)	0.111 (0.006)	0.366 (0.015)	0.231 (0.011)
$\hookrightarrow h_x$	0.589 (0.007)	0.113 (0.002)	0.370 (0.006)	0.203 (0.004)	0.577 (0.017)	0.111 (0.006)	0.366 (0.015)	0.230 (0.011)
$\hookrightarrow \alpha = 1$	0.585 (0.004)	0.114 (0.002)	0.371 (0.006)	0.203 (0.004)	0.576 (0.017)	0.112 (0.006)	0.367 (0.016)	0.231 (0.011)
$\hookrightarrow \alpha = 5$	0.592 (0.009)	0.113 (0.002)	0.369 (0.005)	0.203 (0.004)	0.577 (0.019)	0.111 (0.006)	0.365 (0.014)	0.231 (0.010)
$\hookrightarrow \alpha = 10$	0.592 (0.010)	0.113 (0.002)	0.370 (0.005)	0.204 (0.004)	0.576 (0.016)	0.111 (0.006)	0.365 (0.015)	0.231 (0.010)
$\hookrightarrow \beta = 0.05$	0.591 (0.008)	0.113 (0.002)	0.369 (0.006)	0.203 (0.004)	0.576 (0.018)	0.111 (0.006)	0.365 (0.015)	0.231 (0.010)
$\hookrightarrow \beta = 0.2$	0.582 (0.004)	0.114 (0.002)	0.372 (0.006)	0.203 (0.004)	0.575 (0.017)	0.112 (0.006)	0.368 (0.015)	0.231 (0.011)
$\hookrightarrow \gamma = 0.05$	0.590 (0.006)	0.113 (0.002)	0.371 (0.005)	0.203 (0.004)	0.579 (0.019)	0.111 (0.006)	0.366 (0.015)	0.231 (0.011)
$\hookrightarrow \gamma = 0.2$	0.591 (0.007)	0.114 (0.003)	0.371 (0.006)	0.205 (0.004)	0.576 (0.012)	0.112 (0.006)	0.367 (0.016)	0.232 (0.011)
$\hookrightarrow$ dim S	0.590 (0.006)	0.114 (0.003)	0.371 (0.007)	0.204 (0.005)	0.580 (0.016)	0.112 (0.006)	0.367 (0.016)	0.231 (0.011)
$\hookrightarrow$ dim L	0.587 (0.003)	0.114 (0.002)	0.372 (0.005)	0.205 (0.003)	0.579 (0.015)	0.112 (0.006)	0.368 (0.014)	0.234 (0.010)

Chronic Kidney Disease

Experiment	Baseline				Distribution Shift			
	C^{td} $\uparrow$	IBS $\downarrow$	INBLL $\downarrow$	-Likelihood $\downarrow$	C^{td} $\uparrow$	IBS $\downarrow$	INBLL $\downarrow$	-Likelihood $\downarrow$
MissCVAE	0.787 (0.006)	0.137 (0.002)	0.423 (0.004)	0.198 (0.009)	0.762 (0.002)	0.143 (0.001)	0.438 (0.003)	0.273 (0.008)
$\hookrightarrow h_x$	0.787 (0.005)	0.137 (0.001)	0.423 (0.003)	0.195 (0.009)	0.762 (0.003)	0.143 (0.002)	0.439 (0.004)	0.273 (0.005)
$\hookrightarrow \alpha = 1$	0.786 (0.005)	0.136 (0.002)	0.423 (0.004)	0.202 (0.003)	0.761 (0.001)	0.143 (0.000)	0.438 (0.001)	0.278 (0.001)
$\hookrightarrow \alpha = 5$	0.787 (0.006)	0.136 (0.002)	0.423 (0.005)	0.200 (0.004)	0.762 (0.001)	0.143 (0.001)	0.439 (0.001)	0.279 (0.002)
$\hookrightarrow \alpha = 10$	0.787 (0.005)	0.136 (0.002)	0.423 (0.005)	0.188 (0.017)	0.761 (0.003)	0.144 (0.001)	0.441 (0.003)	0.268 (0.013)
$\hookrightarrow \beta = 0.05$	0.787 (0.006)	0.137 (0.002)	0.423 (0.004)	0.201 (0.003)	0.763 (0.001)	0.142 (0.000)	0.437 (0.001)	0.277 (0.001)
$\hookrightarrow \beta = 0.2$	0.786 (0.005)	0.136 (0.002)	0.423 (0.004)	0.199 (0.007)	0.761 (0.002)	0.143 (0.001)	0.439 (0.002)	0.277 (0.003)
$\hookrightarrow \gamma = 0.05$	0.787 (0.005)	0.136 (0.002)	0.423 (0.004)	0.197 (0.009)	0.761 (0.003)	0.143 (0.001)	0.439 (0.002)	0.275 (0.009)
$\hookrightarrow \gamma = 0.2$	0.786 (0.005)	0.137 (0.002)	0.424 (0.004)	0.202 (0.004)	0.762 (0.001)	0.142 (0.000)	0.437 (0.001)	0.277 (0.001)
$\hookrightarrow$ dim S	0.785 (0.005)	0.137 (0.002)	0.424 (0.004)	0.191 (0.012)	0.760 (0.002)	0.144 (0.001)	0.440 (0.002)	0.269 (0.011)
$\hookrightarrow$ dim L	0.785 (0.007)	0.137 (0.003)	0.424 (0.006)	0.197 (0.003)	0.760 (0.001)	0.144 (0.001)	0.441 (0.002)	0.277 (0.005)

Chronic Kidney Disease – Unseen Missingness Patterns

Experiment	Full Test Set				Subset of Unseen Patterns Only			
	C^{td} $\uparrow$	IBS $\downarrow$	INBLL $\downarrow$	-Likelihood $\downarrow$	C^{td} $\uparrow$	IBS $\downarrow$	INBLL $\downarrow$	-Likelihood $\downarrow$
MissCVAE	0.785 (0.004)	0.137 (0.001)	0.426 (0.003)	0.203 (0.002)	0.781 (0.006)	0.142 (0.002)	0.437 (0.007)	0.209 (0.012)
$\hookrightarrow h_x$	0.784 (0.005)	0.138 (0.001)	0.426 (0.003)	0.203 (0.003)	0.779 (0.007)	0.143 (0.002)	0.440 (0.005)	0.210 (0.013)
$\hookrightarrow \alpha = 1$	0.783 (0.003)	0.138 (0.001)	0.426 (0.003)	0.205 (0.002)	0.779 (0.006)	0.142 (0.002)	0.438 (0.006)	0.211 (0.012)
$\hookrightarrow \alpha = 5$	0.785 (0.004)	0.138 (0.001)	0.427 (0.003)	0.203 (0.003)	0.780 (0.006)	0.142 (0.002)	0.439 (0.006)	0.209 (0.014)
$\hookrightarrow \alpha = 10$	0.786 (0.004)	0.138 (0.001)	0.426 (0.003)	0.203 (0.003)	0.781 (0.006)	0.142 (0.002)	0.438 (0.007)	0.209 (0.014)
$\hookrightarrow \beta = 0.05$	0.786 (0.004)	0.137 (0.001)	0.426 (0.003)	0.203 (0.003)	0.781 (0.005)	0.142 (0.002)	0.437 (0.007)	0.209 (0.013)
$\hookrightarrow \beta = 0.2$	0.784 (0.003)	0.138 (0.001)	0.427 (0.003)	0.204 (0.003)	0.780 (0.006)	0.142 (0.002)	0.438 (0.006)	0.210 (0.014)
$\hookrightarrow \gamma = 0.05$	0.785 (0.004)	0.138 (0.001)	0.426 (0.003)	0.204 (0.003)	0.781 (0.007)	0.142 (0.002)	0.438 (0.006)	0.209 (0.014)
$\hookrightarrow \gamma = 0.2$	0.784 (0.004)	0.138 (0.001)	0.426 (0.002)	0.205 (0.002)	0.781 (0.006)	0.142 (0.002)	0.438 (0.006)	0.210 (0.012)
$\hookrightarrow$ dim S	0.784 (0.004)	0.137 (0.001)	0.425 (0.003)	0.204 (0.002)	0.779 (0.007)	0.142 (0.002)	0.438 (0.007)	0.211 (0.011)
$\hookrightarrow$ dim L	0.786 (0.004)	0.137 (0.002)	0.426 (0.003)	0.202 (0.005)	0.782 (0.008)	0.141 (0.002)	0.437 (0.007)	0.208 (0.014)

All-Cause Mortality

Experiment	Baseline				Distribution Shift			
	C^{td} ↑	IBS ↓	INBLL ↓	-Likelihood ↓	C^{td} ↑	IBS ↓	INBLL ↓	-Likelihood ↓
MissCVAE	0.715 (0.007)	0.058 (0.001)	0.210 (0.005)	0.315 (0.008)	0.707 (0.002)	0.069 (0.000)	0.244 (0.001)	0.377 (0.001)
$\hookrightarrow h_x$	0.709 (0.008)	0.058 (0.001)	0.211 (0.004)	0.315 (0.008)	0.703 (0.003)	0.070 (0.000)	0.246 (0.001)	0.380 (0.001)
$\hookrightarrow \alpha = 1$	0.689 (0.009)	0.061 (0.001)	0.226 (0.002)	0.341 (0.004)	0.686 (0.004)	0.072 (0.000)	0.257 (0.002)	0.398 (0.002)
$\hookrightarrow \alpha = 5$	0.716 (0.007)	0.057 (0.001)	0.209 (0.004)	0.313 (0.007)	0.711 (0.002)	0.069 (0.000)	0.243 (0.001)	0.374 (0.002)
$\hookrightarrow \alpha = 10$	0.717 (0.007)	0.057 (0.001)	0.209 (0.004)	0.312 (0.007)	0.712 (0.001)	0.069 (0.000)	0.242 (0.000)	0.373 (0.000)
$\hookrightarrow \beta = 0.05$	0.715 (0.007)	0.058 (0.001)	0.211 (0.004)	0.316 (0.008)	0.709 (0.003)	0.069 (0.000)	0.244 (0.001)	0.378 (0.001)
$\hookrightarrow \beta = 0.2$	0.701 (0.008)	0.059 (0.001)	0.216 (0.005)	0.324 (0.008)	0.697 (0.008)	0.070 (0.001)	0.249 (0.005)	0.384 (0.008)
$\hookrightarrow \gamma = 0.05$	0.715 (0.006)	0.058 (0.001)	0.210 (0.004)	0.314 (0.007)	0.711 (0.002)	0.069 (0.000)	0.243 (0.000)	0.375 (0.001)
$\hookrightarrow \gamma = 0.2$	0.691 (0.012)	0.060 (0.001)	0.224 (0.006)	0.339 (0.010)	0.688 (0.011)	0.072 (0.002)	0.256 (0.007)	0.396 (0.010)
$\hookrightarrow$ dim S	0.710 (0.007)	0.058 (0.001)	0.211 (0.004)	0.315 (0.008)	0.706 (0.002)	0.069 (0.000)	0.244 (0.001)	0.377 (0.001)
$\hookrightarrow$ dim L	0.717 (0.007)	0.058 (0.001)	0.211 (0.004)	0.316 (0.007)	0.709 (0.002)	0.070 (0.000)	0.245 (0.001)	0.379 (0.001)

All-Cause Mortality – Unseen Missingness Patterns

Experiment	Full Test Set				Subset of Unseen Patterns Only			
	C^{td} ↑	IBS ↓	INBLL ↓	-Likelihood ↓	C^{td} ↑	IBS ↓	INBLL ↓	-Likelihood ↓
MissCVAE	0.699 (0.014)	0.060 (0.002)	0.219 (0.008)	0.331 (0.012)	0.701 (0.014)	0.057 (0.001)	0.213 (0.003)	0.334 (0.007)
$\hookrightarrow h_x$	0.706 (0.006)	0.058 (0.001)	0.213 (0.004)	0.319 (0.007)	0.703 (0.014)	0.056 (0.002)	0.207 (0.006)	0.324 (0.007)
$\hookrightarrow \alpha = 1$	0.687 (0.008)	0.061 (0.001)	0.227 (0.003)	0.342 (0.005)	0.690 (0.016)	0.058 (0.002)	0.218 (0.005)	0.340 (0.006)
$\hookrightarrow \alpha = 5$	0.712 (0.008)	0.058 (0.001)	0.211 (0.004)	0.316 (0.007)	0.710 (0.015)	0.055 (0.002)	0.203 (0.007)	0.317 (0.008)
$\hookrightarrow \alpha = 10$	0.713 (0.008)	0.058 (0.001)	0.210 (0.004)	0.315 (0.007)	0.710 (0.013)	0.055 (0.002)	0.203 (0.006)	0.316 (0.007)
$\hookrightarrow \beta = 0.05$	0.708 (0.012)	0.059 (0.002)	0.215 (0.006)	0.324 (0.010)	0.706 (0.016)	0.056 (0.002)	0.209 (0.007)	0.327 (0.008)
$\hookrightarrow \beta = 0.2$	0.691 (0.010)	0.060 (0.001)	0.223 (0.003)	0.336 (0.005)	0.694 (0.012)	0.058 (0.002)	0.216 (0.006)	0.337 (0.009)
$\hookrightarrow \gamma = 0.05$	0.708 (0.011)	0.058 (0.002)	0.213 (0.006)	0.319 (0.010)	0.706 (0.018)	0.055 (0.002)	0.206 (0.008)	0.320 (0.010)
$\hookrightarrow \gamma = 0.2$	0.687 (0.017)	0.060 (0.002)	0.224 (0.008)	0.337 (0.014)	0.691 (0.019)	0.057 (0.001)	0.215 (0.002)	0.335 (0.007)
$\hookrightarrow$ dim S	0.705 (0.006)	0.058 (0.001)	0.213 (0.004)	0.319 (0.007)	0.705 (0.014)	0.056 (0.002)	0.205 (0.006)	0.320 (0.006)
$\hookrightarrow$ dim L	0.700 (0.007)	0.060 (0.001)	0.221 (0.003)	0.334 (0.006)	0.697 (0.014)	0.058 (0.001)	0.216 (0.005)	0.339 (0.004)

Table 12: MissCVAE real-world experiments ablation study results.

G Survival Curves

We investigate how different missingness patterns impact survival predictions for the same individual. We use three examples: an unwell individual (latent health = -1), a healthy individual (+1), and an average individual (0), generating risk factors accordingly. We then introduce missingness by masking subsets of features and feed these incomplete data points through trained models to observe how predictions shift. Table 13 presents an example of an unwell individual with several missingness patterns, alongside the corresponding imputed values obtained via iterative imputation. The results show that imputed values can vary drastically especially for outliers and depending on which features are observed.

Table 13: Example of iterative imputation and scaling applied to a single 'unwell' individual (latent health = -1), shown under different missingness patterns across all Simulations.

Induced Missingness

Age	Prior Condition	Treatment	v1	v2	v3	v4	v5	v6	v7	v8	v9	v10	v11	v12	v13	v14	v15
60	1	1	NA	NA	NA	NA	NA	NA	NA	NA	NA	NA	NA	NA	NA	NA	NA
60	1	1	175.0	NA	NA	NA	NA	NA	NA	NA	NA	NA	NA	NA	NA	NA	NA
60	1	1	NA	40.0	150.0	NA	NA	NA	NA	NA	NA	NA	NA	NA	NA	NA	NA
60	1	1	175.0	40.0	150.0	NA	NA	NA	NA	NA	NA	NA	NA	NA	NA	NA	NA
60	1	1	175.0	40.0	150.0	6.5	67.0	6.11	NA	NA	NA	NA	NA	NA	NA	NA	NA
60	1	1	175.0	40.0	150.0	6.5	67.0	6.11	23.0	4.0	6.5	NA	NA	NA	NA	NA	NA
60	1	1	175.0	40.0	150.0	6.5	67.0	6.11	23.0	4.0	6.5	1.3	NA	NA	NA	NA	NA
60	1	1	175.0	40.0	150.0	6.5	67.0	6.11	23.0	4.0	6.5	1.3	410.0	12.0	1.95	0.0	NA
60	1	1	175.0	40.0	150.0	6.5	67.0	6.11	23.0	4.0	6.5	1.3	410.0	12.0	1.95	0.0	3.05

Simulation A (Baseline)

Age	Prior Condition	Treatment	v1	v2	v3	v4	v5	v6	v7	v8	v9	v10	v11	v12	v13	v14	v15
1.71	1	1	0.86	0.42	0.69	0.29	-1.36	0.35	-0.22	0.39	0.30	-0.21	0.35	0.31	0.41	0.18	0.10
1.71	1	1	0.83	0.43	0.69	0.29	-1.36	0.35	-0.22	0.39	0.30	-0.21	0.35	0.31	0.40	0.20	0.10
1.71	1	1	0.87	1.85	2.74	0.90	-1.77	1.12	-0.95	1.40	0.88	-0.75	0.62	0.80	0.35	0.39	0.16
1.71	1	1	0.83	1.85	2.74	0.90	-1.77	1.12	-0.95	1.41	0.88	-0.76	0.61	0.81	0.34	0.42	0.16
1.71	1	1	0.83	1.85	2.74	1.15	-1.93	1.46	-1.01	1.43	0.90	-0.83	0.58	0.87	0.34	0.54	0.22
1.71	1	1	0.83	1.85	2.74	1.15	-1.93	1.46	-0.77	1.64	0.81	-0.90	0.65	0.73	0.35	0.55	0.23
1.71	1	1	0.83	1.85	2.74	1.15	-1.93	1.46	-0.77	1.64	0.81	-0.68	0.64	1.15	0.36	0.52	0.23
1.71	1	1	0.83	1.85	2.74	1.15	-1.93	1.46	-0.77	1.64	0.81	-0.68	0.67	1.20	-0.26	-2.05	0.22
1.71	1	1	0.83	1.85	2.74	1.15	-1.93	1.46	-0.77	1.64	0.81	-0.68	0.67	1.20	-0.26	-2.05	0.29

Simulation B (Unseen Patterns)

Age	Prior Condition	Treatment	v1	v2	v3	v4	v5	v6	v7	v8	v9	v10	v11	v12	v13	v14	v15
1.78	1	1	0.86	0.41	0.67	0.30	-1.43	0.36	-0.09	0.46	0.28	-0.25	0.05	0.42	0.27	0.32	0.02
1.78	1	1	0.84	0.41	0.67	0.30	-1.44	0.36	-0.10	0.47	0.28	-0.26	0.05	0.43	0.27	0.33	0.02
1.78	1	1	0.88	1.94	2.91	0.93	-1.86	1.17	-0.68	1.69	0.83	-0.95	0.42	1.21	0.14	0.56	0.19
1.78	1	1	0.84	1.94	2.91	0.93	-1.86	1.17	-0.69	1.69	0.82	-0.96	0.42	1.22	0.14	0.58	0.19
1.78	1	1	0.84	1.94	2.91	1.26	-2.02	1.55	-0.70	1.65	0.86	-1.05	0.31	1.30	0.17	0.70	0.21
1.78	1	1	0.84	1.94	2.91	1.26	-2.02	1.55	-0.96	2.00	0.84	-0.94	0.21	1.46	0.42	0.78	0.20
1.78	1	1	0.84	1.94	2.91	1.26	-2.02	1.55	-0.96	2.00	0.84	-0.85	0.20	1.47	0.25	0.77	0.20
1.78	1	1	0.84	1.94	2.91	1.26	-2.02	1.55	-0.96	2.00	0.84	-0.85	0.98	1.53	-0.25	-1.81	14.79
1.78	1	1	0.84	1.94	2.91	1.26	-2.02	1.55	-0.96	2.00	0.84	-0.85	0.98	1.53	-0.25	-1.81	0.32

Simulation C (Missingness Shift)

Age	Prior Condition	Treatment	v1	v2	v3	v4	v5	v6	v7	v8	v9	v10	v11	v12	v13	v14	v15
1.71	1	1	0.87	0.45	0.68	0.32	-1.34	0.40	-0.19	0.29	0.22	-0.10	0.12	0.17	-0.51	0.31	0.07
1.71	1	1	0.83	0.45	0.68	0.32	-1.34	0.42	-0.20	0.29	0.22	-0.10	0.12	0.17	-0.51	0.32	0.06
1.71	1	1	0.85	1.89	2.75	0.95	-1.72	1.12	-0.49	0.91	0.54	-0.73	0.35	0.65	-0.08	1.60	0.23
1.71	1	1	0.83	1.89	2.75	0.96	-1.72	1.14	-0.48	0.91	0.55	-0.73	0.35	0.65	-0.08	1.61	0.23
1.71	1	1	0.83	1.89	2.75	1.22	-1.90	1.42	-0.49	0.96	0.56	-0.66	0.35	0.77	-0.08	1.49	0.26
1.71	1	1	0.83	1.89	2.75	1.22	-1.90	1.42	-0.54	1.27	0.65	-0.68	0.37	0.86	0.00	1.41	0.28
1.71	1	1	0.83	1.89	2.75	1.22	-1.90	1.42	-0.54	1.27	0.65	-0.64	0.41	0.85	0.03	1.41	0.28
1.71	1	1	0.83	1.89	2.75	1.22	-1.90	1.42	-0.54	1.27	0.65	-0.64	0.46	1.02	-0.05	-1.02	-8.49
1.71	1	1	0.83	1.89	2.75	1.22	-1.90	1.42	-0.54	1.27	0.65	-0.64	0.46	1.02	-0.05	-1.02	0.32

Figure 2 shows survival predictions across different missingness patterns. Across all models, predictions for fully missing inputs (pattern of all 0s) tend to underestimate risk for the “unwell” individual and overestimate risk for the “well” individual. Among models that incorporate missingness information, MissCVAE exhibits the smallest variation in predictions across patterns, suggesting greater robustness and reduced overfitting to missingness signals (notably Figure 2b).

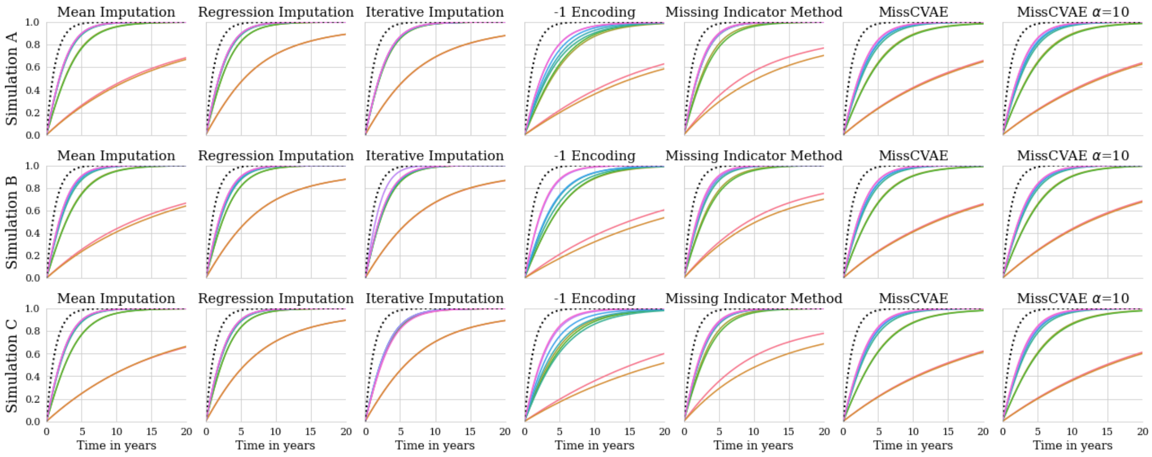

(a) Predictions for a single ‘unwell’ individual (latent health = -1).

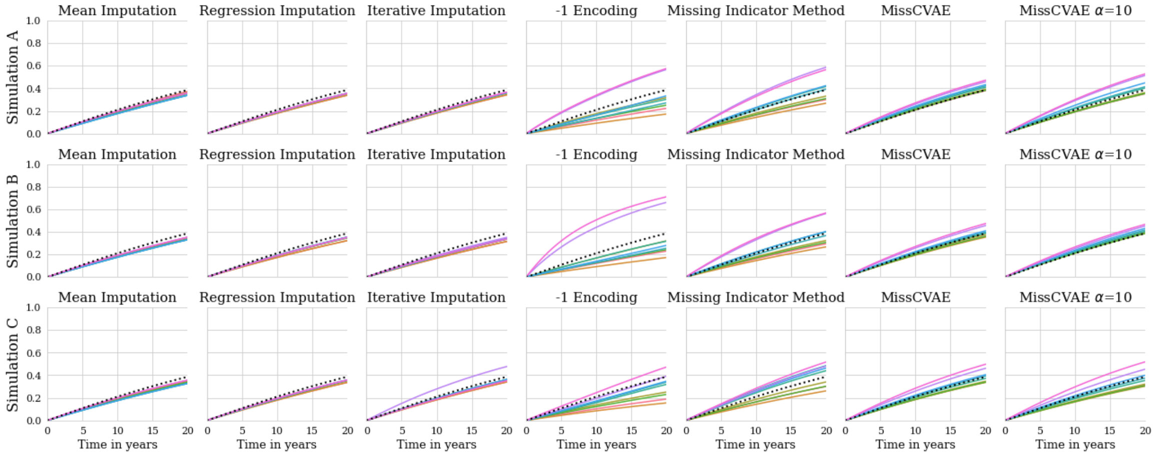

(b) Predictions for a single ‘average’ individual (latent health = 0)

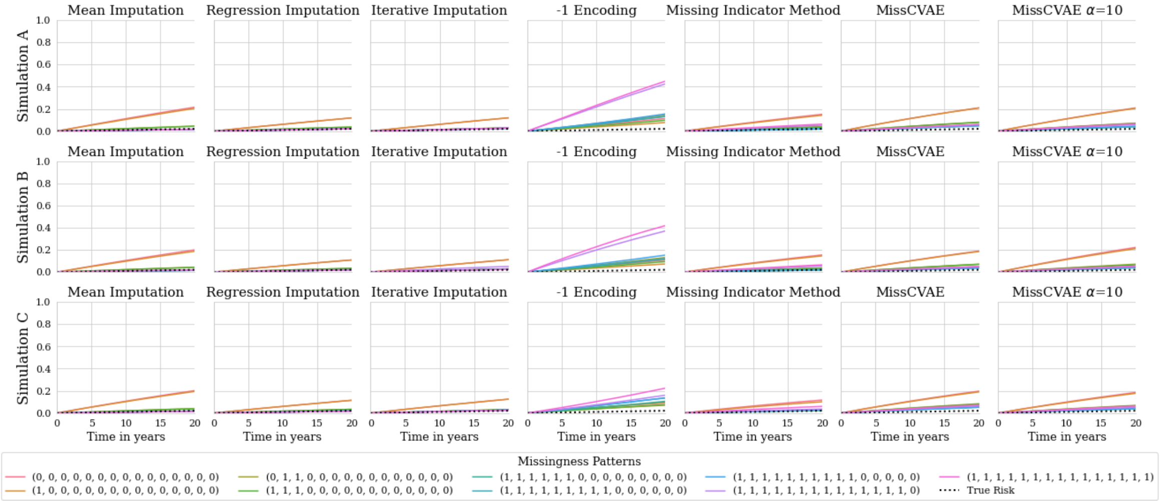

(c) Predictions for a single ‘healthy’ individual (latent health = +1)

Figure 2: Absolute risk predictions across a set of missingness patterns in Simulations.

H Time-Dependent Calibration Plots

Figures 3–6 present time-dependent discrete calibration plots for all models. We evaluate calibration at two time points: a short-term horizon of 5 years and a long-term horizon equal to the maximum observed follow-up time in each dataset (20 years for simulations, 17.5 years for CKD and CVD, and 15 years for All-Cause Mortality).

Since true event probabilities are inherently unobservable in practice, we estimate observed event probabilities by grouping individuals into bins and computing empirical proportions within each bin. The standard approach partitions individuals based on quantiles of their predicted risk. Calibration plots are constructed as follows:

1. Select an evaluation time point t^*.
2. For each individual, compute the predicted risk at t^*:

$$\hat{p}_i = \Pr(T \leq t^* \mid \tilde{\mathbf{x}}_i, \mathbf{m}_i) = 1 - S(t^* \mid \tilde{\mathbf{x}}_i, \mathbf{m}_i).$$

3. Partition individuals into B bins according to quantiles of $\hat{p}_i$ (here, $B = 10$ deciles).
4. For each bin b:
   (a) Fit a Kaplan–Meier estimator using only individuals in that bin to obtain the empirical survival curve $\hat{S}_b(t)$;
   (b) Evaluate the survival curve at t^* to obtain the estimated observed event probability:

$$\hat{o}_b = 1 - \hat{S}_b(t^*).$$

5. For each bin, plot the observed risk $\hat{o}_b$ against the mean predicted risk $\bar{p}_b$ to obtain the discrete calibration plot.

To numerically quantify calibration, we report the Expected Calibration Error (ECE) at time t^* (Tables 14–17), defined as

$$\mathrm{ECE}(t^*) = \sum_{b=1}^{B} \frac{n_b}{n} \left| \hat{o}_b - \bar{p}_b \right|,$$

where n_b is the number of individuals in bin b, $\hat{o}_b$ is the observed event probability at t^* obtained from the Kaplan–Meier curve for that bin, and $\bar{p}_b$ is the mean predicted risk in that bin.

Results. Across most datasets and time horizons, all models lie close to the 45-degree perfect calibration line. We observe a general pattern where at short horizons, lower-risk deciles tend to be better calibrated (e.g., All-Cause Mortality shows miscalibration in higher-risk deciles at 5 years), while at longer horizons, mid-to-high risk deciles exhibit better calibration. The CVD dataset is the exception, as most observations have predicted risks below 0.5 and are clustered in lower-risk bins, even at longer horizons. All models systematically underestimate risks in the 0.5–0.6 range, with no predictions exceeding 0.6. Visually, all models appear similarly calibrated, suggesting that this approach has limited ability to distinguish miscalibration patterns.

These plots serve primarily as a sanity check rather than a tool for model comparison. In the simulations, even the ground truth model does not consistently outperform benchmarks based on ECE. However, in informative real-world datasets (CKD and All-Cause Mortality), the proposed model frequently achieves the smallest ECE, particularly at longer time horizons. Because ECE is not a proper scoring rule and these plots are primarily diagnostic, we present results from a single fold rather than reporting means and standard deviations across all five folds.

Model	Simulation A		Simulation B		Simulation C	
	**5 Year**	**20 Year**	**5 Year**	**20 Year**	**5 Year**	**20 Year**
Mean	0.0109	**0.0108**	**0.0173**	0.0408	**0.0055**	0.0204
RI	**0.0066**	0.0143	0.0203	0.0453	0.0068	0.0216
ICE	0.0077	0.0228	0.0410	0.0579	0.0075	0.0199
-1 Enc.	0.0099	0.0246	0.0264	**0.0359**	0.0198	0.0256
MIM	0.0109	0.0218	0.0211	0.0572	0.0118	0.0276
MissCVAE	0.0168	0.0296	0.0308	0.0363	0.0156	0.0312
Truth	**0.0066**	0.0184	0.0409	0.0507	0.0066	**0.0184**

Table 14: Expected Calibration Error (ECE) in Simulations at 5-year and 20-year horizons.

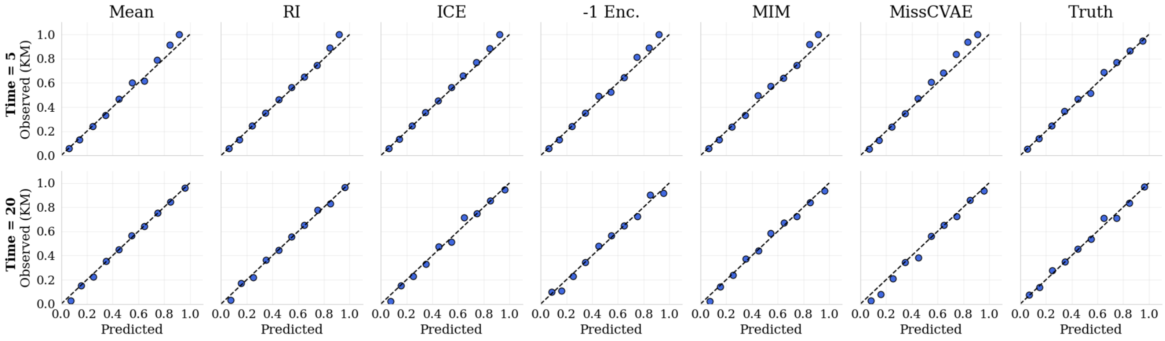

(a) Simulation A (Baseline)

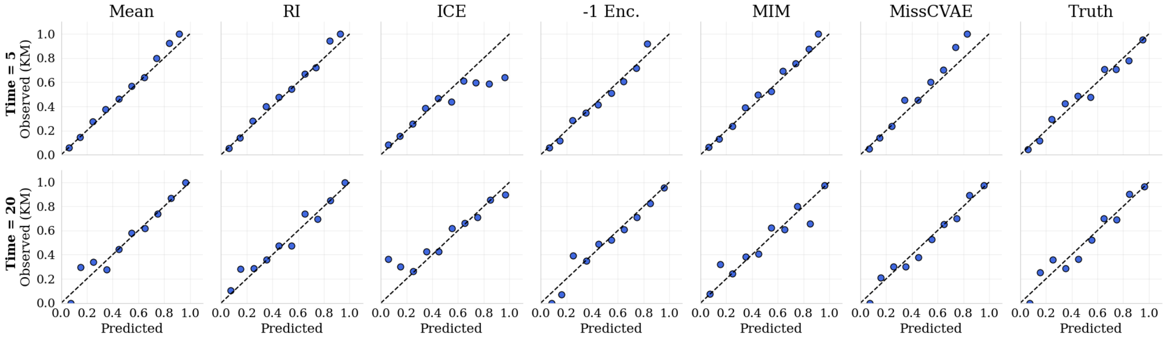

(b) Simulation B (Unseen Patterns)

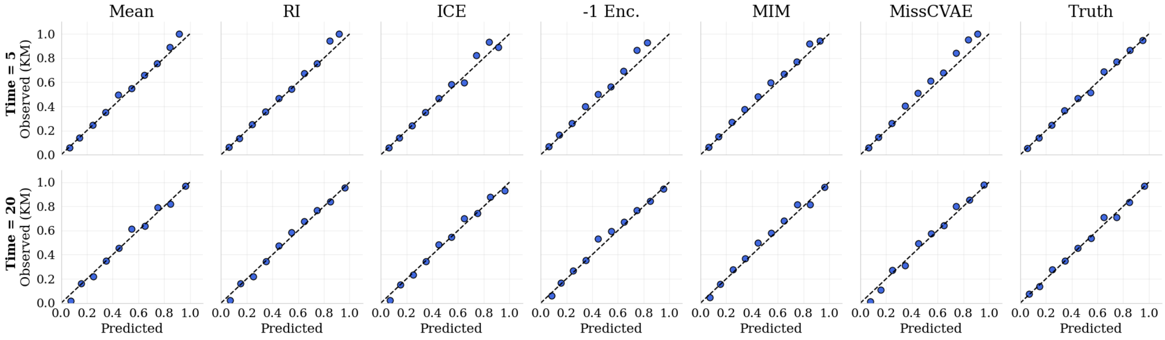

(c) Simulation C (Missingness Shift)

Figure 3: Discrete calibration plots based on predicted-risk deciles from the simulation studies, showing calibration at 5-year and 20-year risk.

Model	CKD (Baseline)		CKD (Unseen)		CKD (Shift)	
	**5 Year**	**17.5 Year**	**5 Year**	**17.5 Year**	**5 Year**	**17.5 Year**
Mean	0.0208	0.0244	0.0306	0.0602	0.0244	0.0699
RI	0.0225	0.0313	0.0314	0.0660	0.0236	0.0672
ICE	0.0207	0.0209	0.0356	0.0570	0.0236	0.0668
-1 Enc.	0.0188	0.0238	0.0262	0.0622	**0.0220**	0.0501
MIM	0.0229	0.0303	**0.0155**	0.0428	0.0270	0.0687
MissCVAE	**0.0183**	**0.0158**	0.0212	**0.0425**	0.0263	**0.0337**

Table 15: Expected Calibration Error (ECE) in CKD at 5-year and 20-year horizons.

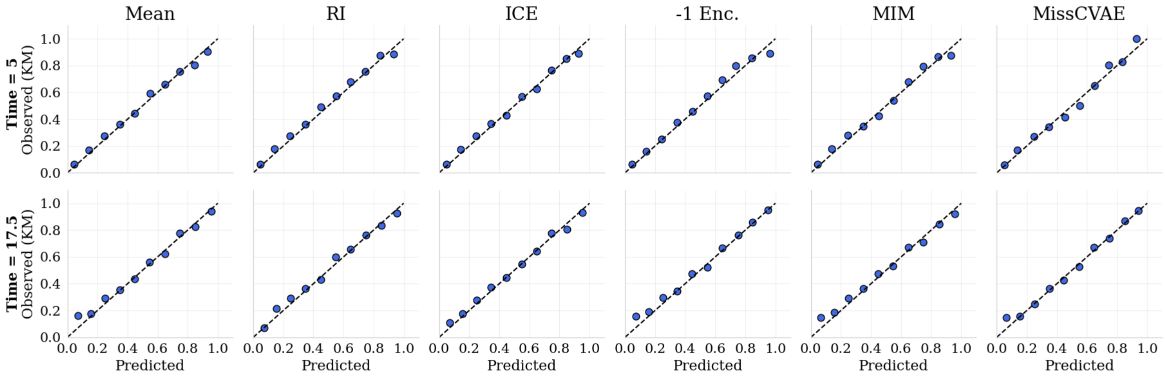

(a) CKD (Baseline)

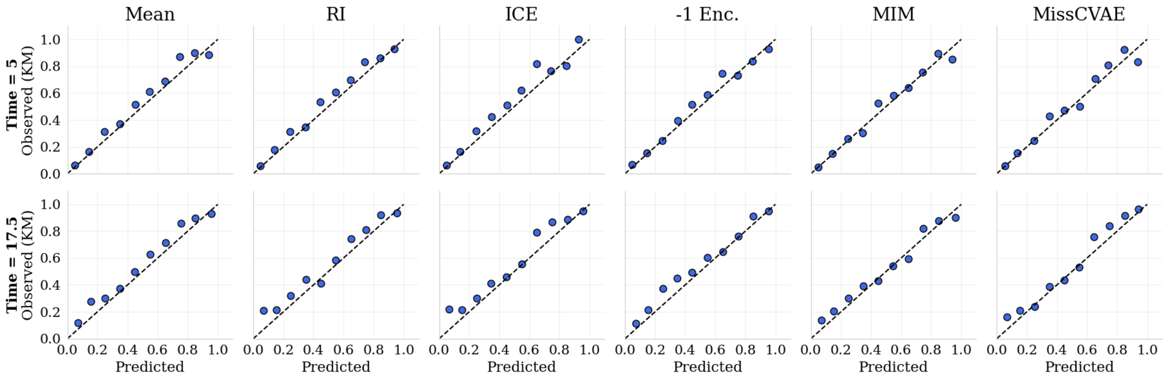

(b) CKD (Unseen Patterns)

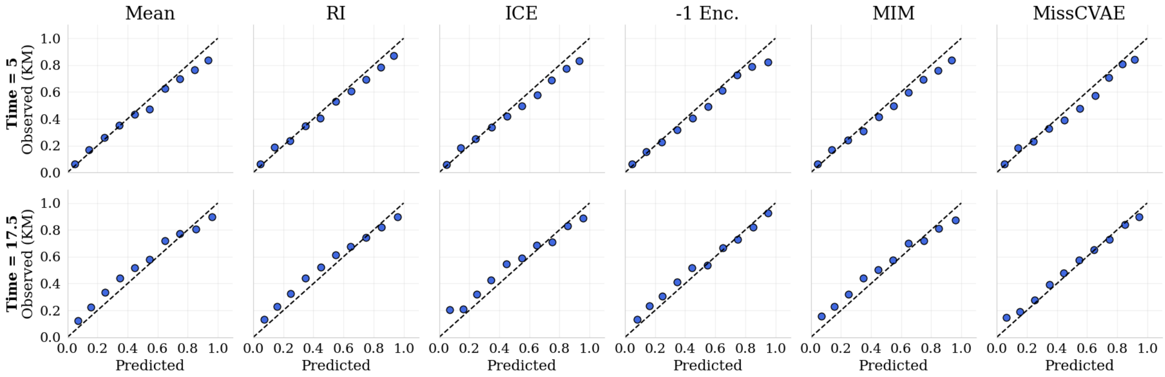

(c) CKD (Distribution Shift)

Figure 4: Discrete calibration plots based on predicted-risk deciles from the CKD experiments, showing calibration at 5-year and 17.5-year risk.

Model	CVD (Baseline)		CVD (Unseen)		CVD (Shift)	
	5 Year	17.5 Year	5 Year	17.5 Year	5 Year	17.5 Year
Mean	0.0079	0.0241	0.0178	**0.0374**	0.0060	0.0362
RI	0.0079	0.0293	0.0179	0.0567	0.0055	0.0434
ICE	0.0081	**0.0235**	0.0178	0.0470	0.0085	0.0364
-1 Enc.	0.0074	0.0276	**0.0125**	0.0708	0.0032	**0.0205**
MIM	**0.0062**	0.0259	0.0147	0.0480	0.0072	0.0289
MissCVAE	0.0145	0.0496	0.0272	0.0674	**0.0023**	0.0278

Table 16: Expected Calibration Error (ECE) in CVD at 5-year and 17.5-year horizons.

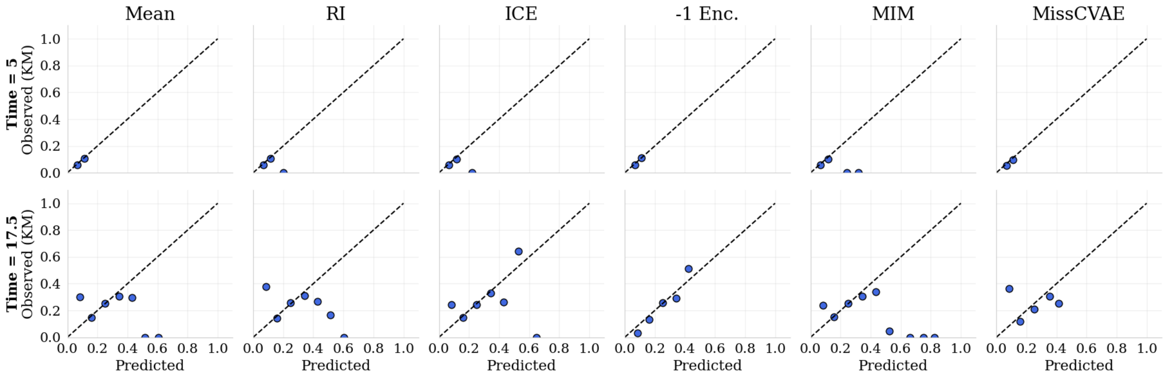

(a) CVD (Baseline)

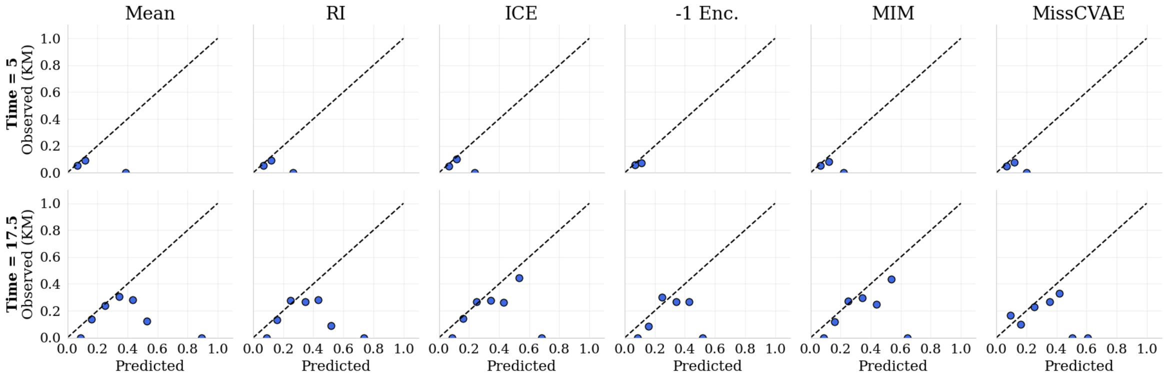

(b) CVD (Unseen Patterns)

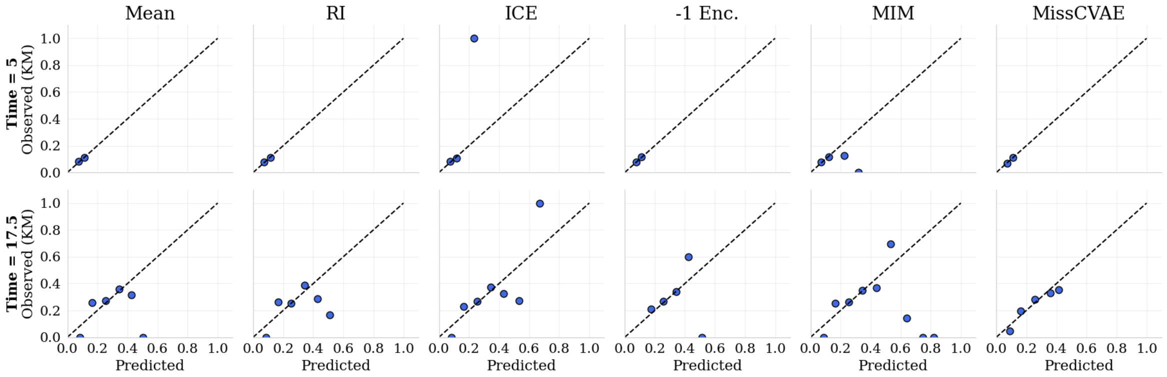

(c) CVD (Distribution Shift)

Figure 5: Discrete calibration plots based on predicted-risk deciles from the CVD experiments, showing calibration at 5-year and 17.5-year risk.

Model	ACM (Baseline)		ACM (Unseen)		ACM (Shift)	
	5 Year	15 Year	5 Year	15 Year	5 Year	15 Year
Mean	0.0179	0.0554	0.0199	0.0661	0.0185	0.0818
RI	0.0193	0.0597	0.0173	0.0734	0.0200	0.0818
ICE	0.0179	0.0564	0.0257	0.0663	0.0197	0.0779
-1 Enc.	0.0185	0.0501	0.0266	0.0801	0.0145	0.0681
MIM	0.0158	0.0565	0.0180	0.0767	0.0184	0.0796
MissCVAE	**0.0140**	**0.0381**	**0.0161**	**0.0447**	**0.0107**	**0.0664**

Table 17: Expected Calibration Error (ECE) in All-Cause Mortality (ACM) at 5-year and 20-year horizons.

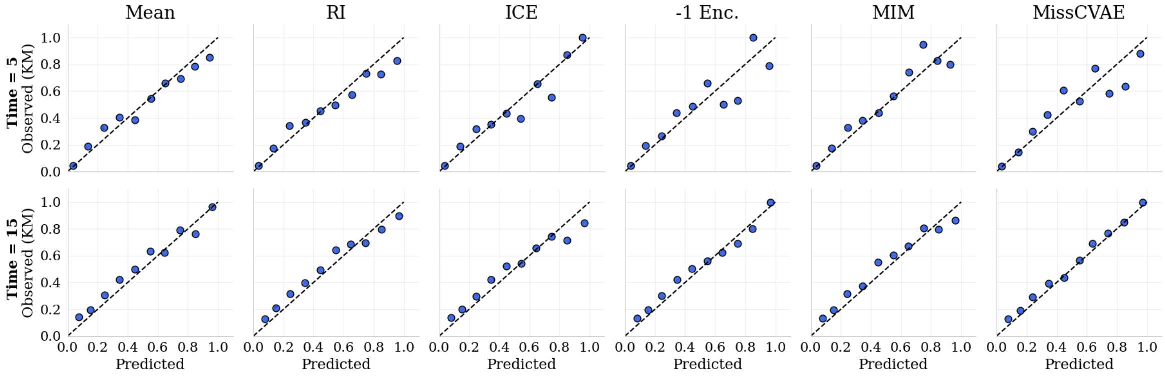

(a) All-Cause Mortality (Baseline)

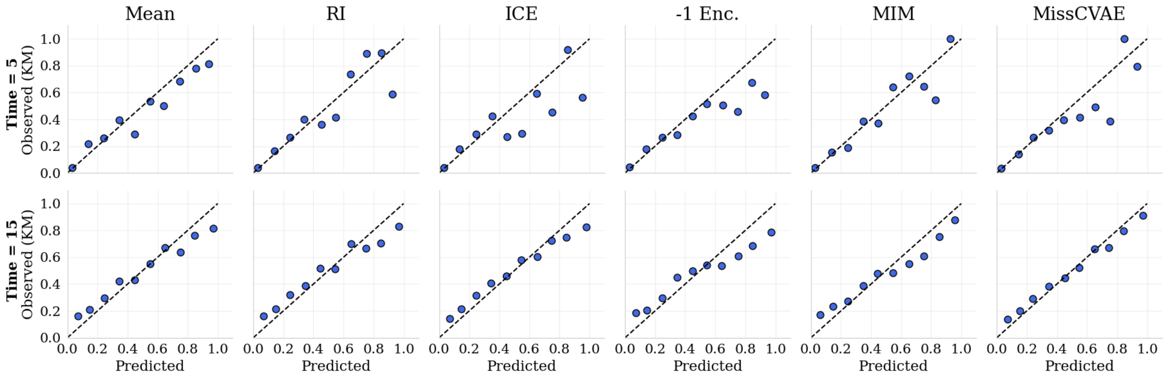

(b) All-Cause Mortality (Unseen Patterns)

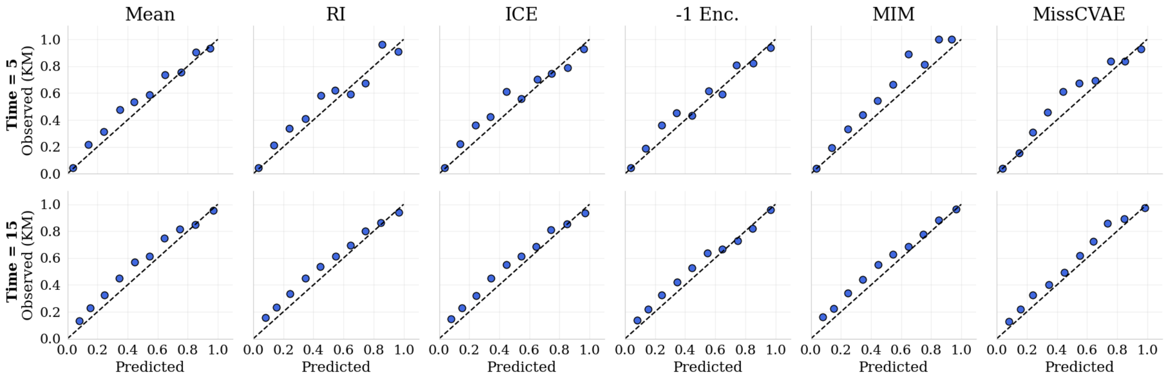

(c) All-Cause Mortality (Distribution Shift)

Figure 6: Discrete calibration plots based on predicted-risk deciles from the All-Cause Mortality experiments, showing calibration at 5-year and 15-year risk.

I Resource Usage Analysis

Table 18 summarises resource usage for all methods on a representative fold (fold 0). All experiments were conducted on CPU only via a shared HPC cluster (Intel Xeon Platinum 8360Y, SLURM, 8 cores, 64 GB RAM allocation, no GPU). Peak training RAM, measured via the Weights & Biases RSS metric, was 0.48–0.49 GB across all methods, largely attributable to Python and PyTorch framework overhead. The 64 GB allocation was chosen to accommodate survival inference over dense time grids. In practice, batched data loaders mitigate this cost.

For imputation baselines, imputation and training times are reported separately. For MICE, we additionally report the test-time re-fit imputation cost in the inference column. MissCVAE handles missingness natively, requiring no separate imputation step. The additional VAE components do not meaningfully increase memory or runtime, with scalability advantage deriving from eliminating up to 2^p pattern-specific submodels rather than from differences in raw speed.

Experiment	Method	Imputation Time (s)	Training Time (min)	Inference Time per 10k (s)	Peak Training RAM (GB)
**Simulation A**	CW	–	9.4	8.7	0.476
	CW + M	–	5.5	8.4	0.469
	Mean	0.4	4.9	7.7	0.474
	ICE (iter = 10)[a]	2.0	4.9	6.3	0.469
	RI	0.6	7.0	7.7	0.471
	MICE O ($m = 1$)[b]	10.6	5.4	re-fit imputation: 15.2<br>inference: 6.8	0.471
	MICE ($m = 1$)[b]	6.5	6.9	re-fit imputation: 8.0<br>inference: 6.4	0.472
	-1 Enc.	0.5	9.1	9.5	0.469
	MIM	0.4	9.7	8.6	0.474
	MissCVAE	–	4.8	6.3	0.475
**CKD**	CW	–	13.2	14.4	0.480
	CW + M	–	13.2	14.5	0.479
	Mean	0.5	5.0	14.6	0.484
	ICE (iter = 10)[a]	90.4	7.8	13.0	0.483
	RI	1.9	9.7	12.7	0.483
	MICE O ($m = 1$)[b]	96.0	7.4	re-fit imputation: 108.5<br>inference: 12.1	0.487
	MICE ($m = 1$)[b]	45.0	5.3	re-fit imputation: 58.4<br>inference: 12.1	0.485
	-1 Enc.	0.5	13.4	11.8	0.489
	MIM	0.5	7.7	13.0	0.486
	MissCVAE	–	13.3	12.1	0.487

[a] sklearn IterativeImputer with default `max_iter`= 10 and `tol`= 10^{-3}. Convergence is not guaranteed; increasing `max_iter` increases imputation time.
[b] MICE time reported for a single imputation ($m = 1$). In practice, multiple imputations are required (default $m = 5$), each requiring a separate downstream model, scaling time linearly.

Table 18: Resource usage on a representative fold (fold 0). Imputation time is reported separately from downstream model training time. Inference time includes any required test-time processing and imputation.

References

[1] D.B. Rubin. Inference and missing data. *Biometrika*, 63(3):581–592, 12 1976.

[2] E. L. Kaplan and Paul Meier. Nonparametric estimation from incomplete observations. *Journal of the American Statistical Association*, 53(282):457–481, 1958.

[3] Laura Antolini, Patrizia Boracchi, and Elia Biganzoli. A time-dependent discrimination index for survival data. *Statistics in Medicine*, 24(24):3927–3944, 2005.

[4] Shona Livingstone, Daniel R. Morales, Peter T. Donnan, Katherine Payne, Alexander J. Thompson, Ji-Hee Youn, and Bruce Guthrie. Effect of competing mortality risks on predictive performance of the QRISK3 cardiovascular risk prediction tool in older people and those with comorbidity: External validation population cohort study. *The Lancet Healthy Longevity*, 2(6):e352–e361, June 2021.

[5] Glenn W. Brier. Verification of forecasts expressed in terms of probability. *Monthly Weather Review*, 78(1):1 – 3, 1950.

[6] Erika Graf, Claudia Schmoor, Willi Sauerbrei, and Martin Schumacher. Assessment and comparison of prognostic classification schemes for survival data. *Statistics in Medicine*, 18(17-18):2529–2545, 1999.

[7] Håvard Kvamme and Ørnulf Borgan. The brier score under administrative censoring: Problems and a solution. *Journal of Machine Learning Research*, 24(2):1–26, 2023.

[8] Håvard Kvamme, Ørnulf Borgan, and Ida Scheel. Time-to-event prediction with neural networks and cox regression. *J. Mach. Learn. Res.*, 20:129:1–129:30, 2019.

[9] Dominic Danks and Christopher Yau. Derivative-based neural modelling of cumulative distribution functions for survival analysis. In Gustau Camps-Valls, Francisco J. R. Ruiz, and Isabel Valera, editors, *Proceedings of The 25th International Conference on Artificial Intelligence and Statistics*, volume 151 of *Proceedings of Machine Learning Research*, pages 7240–7256. PMLR, 28–30 Mar 2022.

[10] Fabian Pedregosa, Gaël Varoquaux, Alexandre Gramfort, Vincent Michel, Bertrand Thirion, Olivier Grisel, Mathieu Blondel, Peter Prettenhofer, Ron Weiss, Vincent Dubourg, et al. Scikit-learn: Machine learning in python. *Journal of machine learning research*, 12(Oct):2825–2830, 2011.

[11] Stef van Buuren and Karin Groothuis-Oudshoorn. mice: Multivariate imputation by chained equations in r. *Journal of Statistical Software*, 45(3):1–67, 2011.

[12] William Falcon and The PyTorch Lightning team. PyTorch Lightning, March 2019.
